# Fontan Circulation Associated Organ Abnormalities Beyond the Heart, Lungs, Liver, and Gut: A Systematic Review

**DOI:** 10.3389/fcvm.2022.826096

**Published:** 2022-03-22

**Authors:** Evi Ritmeester, Veerle A. Veger, Jelle P. G. van der Ven, Gabrielle M. J. W. van Tussenbroek, Carine I. van Capelle, Floris E. A. Udink ten Cate, Willem A. Helbing

**Affiliations:** ^1^Division of Pediatric Cardiology, Department of Pediatrics, Erasmus Medical Center Sophia Children's Hospital, Rotterdam, Netherlands; ^2^Netherlands Heart Institute, Utrecht, Netherlands; ^3^Department of Pediatrics, Erasmus Medical Center Sophia Children's Hospital, Rotterdam, Netherlands; ^4^Department of Pediatric Cardiology, Amalia Children's Hospital, Radboud University Medical Center, Nijmegen, Netherlands

**Keywords:** univentricular congenital heart defects, neurology, renal disease, endocrinology, long term outcomes, metabolism, musculoskeletal system, Fontan circulation

## Abstract

**Introduction:**

Patients with a Fontan circulation are at risk for sequelae of Fontan physiology during follow-up. Fontan physiology affects all organ systems and an overview of end-organ damage is needed.

**Methods:**

We performed a systematic review of abnormalities in multiple organ systems for patients with a longstanding Fontan circulation. We searched online databases for articles describing abnormalities in multiple organ systems. Cardio-pulmonary abnormalities, protein losing enteropathy, and Fontan associated liver disease have already extensively been described and were excluded from this systematic review.

**Results:**

Our search returned 5,704 unique articles. After screening, we found 111 articles relating to multiple organ systems. We found abnormalities in, among others, the nervous system, pituitary, kidneys, and musculoskeletal system. Pituitary edema—relating to the unique pituitary vasculature- may affect the thyroid axis. Renal dysfunction is common. Creatinine based renal function estimates may be inappropriate due to myopenia. Both lean muscle mass and bone mineral density are decreased. These abnormalities in multiple organ systems may be related to Fontan physiology, cyanosis, iatrogenic factors, or lifestyle.

**Conclusions:**

Health care providers should be vigilant for hypothyroidism, visual or hearing deficits, and sleep disordered breathing in Fontan patients. We recommend including cystatin C for assessment of renal function. This review may aid health care providers and guide future research.

**Systematic Review Registration:** https://www.crd.york.ac.uk/prospero/display_record.php?ID=CRD42021232461, PROSPERO, identifier: CRD42021232461.

## Introduction

Patients suffering from univentricular congenital heart defects (CHD), in which only one ventricle is sufficiently developed, are commonly palliated with the Fontan procedure. This series of operations diminishes mixing of deoxygenated and oxygenated blood and reduces volume overload of the single ventricle (1). However, the resulting Fontan circulation, in which the systemic and pulmonary circulation are connected in series, is highly abnormal (2). Although the early and intermediate-term survival of Fontan patients has improved significantly over the last 40 years, end-organ sequelae are common and may affect other organs than the cardiovascular system (3).

As survival has improved, the adult Fontan patient population is rapidly growing. A recent study estimated the prevalence of Fontan patients (in Europe, Oceania, and the USA) will increase from 66 per million in 2020 to 79 per million in 2030, an increase of nearly 20% (4). Furthermore, the proportion of adult patients will grow from 55 to 64% in the same time span (4). Long-term follow up has revealed several complications, where the abnormal Fontan circulation affects different organ systems and may lead to multi-organ failure. Well-known life-threatening Fontan-related complications are plastic bronchitis (PB) and protein losing enteropathy (PLE) (3). Other (end-organ) consequences of the long standing Fontan circulation are not well-characterized, nor fully understood (5).

For adequate management of these patients in the context of their growing life expectancy, it is crucial to gain insight in all possible complications of this highly abnormal circulation. As Fontan associated liver disease, PLE, PB, and thrombo-embolic complications have already been extensively described elsewhere (6–8), the present study aims to add to the current literature by providing a review of abnormalities described in organ systems beyond the heart, lungs, liver, and gut for patients with a longstanding Fontan circulation.

## Methods

### Search Strategy

This systematic review has been registered to PROSPERO prior to search (ID: CRD42021232461). The electronic search was performed using Embase, Medline and the Cochrane Central Register of Controlled Trials. In order to find articles, the search term included terms related to the Fontan procedure and to different types of organ systems. The complete search strategy is supplied in the Supplementary Material. The search was conducted on January 18th 2022. All articles from inception to the search date were considered. All articles describing abnormalities in organ systems in patients with a longstanding Fontan circulation were considered, including case reports, case series, retrospective studies, and prospective studies.

### Exclusion Criteria

Articles were excluded from the study when one or more of the following exclusion criteria were met:

– Articles were not written in English;
– Articles did not at least include a subgroup analysis of patients with a completed Fontan circulation (in the setting of papers reporting on different types of CHD);
– Articles regarding the effects of a Fontan circulation on the lungs, cardiovascular system, liver, thrombo-embolism or PLE;
– Articles detailing only short-term outcomes, i.e., <1 year following Fontan completion;
– Articles not deemed relevant to the research question by the authors' unanimous consensus. These articles are listed in the Supplementary Material;
– Commentaries, reviews, meta-analyses, systematic review, and conference abstracts.

### Data Collection Process

All articles were manually screened for eligibility by one of two authors (ER or VAV). First, titles and abstracts were screened. In case of any doubt, a consensus meeting with 2 or more authors was held. Secondly, the full texts of articles not excluded based on title and abstract were screened by the same observers. If there was doubt whether a study was relevant to the research question in- or exclusion was decided upon in a consensus meeting with JPGvdV and WAH. The articles excluded for this reason are listed in the Supplementary Material.

The included articles were classified based on which organ system(s) were studied in the respective article. Articles may be included in more than 1 category. The full articles were read and information regarding the function of organ systems of Fontan patients was extracted by hand. Due to the scope of this research and the wide variety of outcome measures, we took a narrative approach to describing study findings. All findings that provided insight in potential dysfunction of the organ system was deemed relevant. No formal quality assessment for articles was performed because of the wide variety of study designs included in our search strategy.

Throughout this review, data is presented as “mean ± standard deviation” or as “median [interquartile range],” unless otherwise specified.

## Results

### Search Results

**Figure 1:**
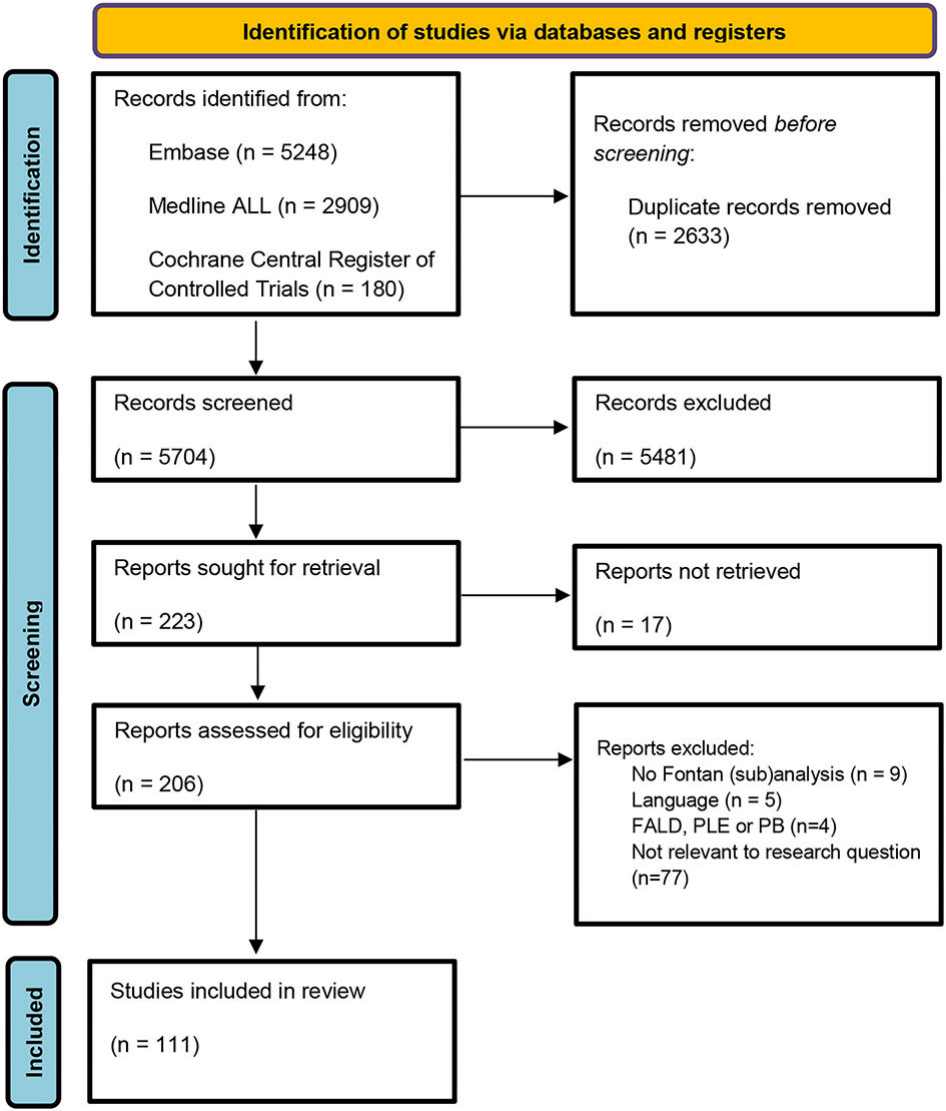
Search strategy flowchart. FALD, Fontan associated liver disease; PLE, protein losing enteropathy; PB, Plastic bronchitis.

Our search returned 5,704 unique publications. The selection process is shown in the flowchart (Figure 1). After completing the assessment for eligibility, 111 studies were included in this systematic review. The publications were sorted into the following groups: neurology (*n* = 32); kidneys (*n* = 19); the muscular system (*n* = 18); endocrinology (*n* = 13); metabolism (*n* = 10); neoplastic disease (*n* = 9); bone (*n* = 9); the immune system (*n* = 5); the auditory system (*n* = 4); reproductive system (*n* = 4); sleep-disordered breathing (*n* = 3); dermatology (*n* = 3); ophthalmology (*n* = 2); dental abnormalities (*n* = 2); gastro-intestinal (*n* = 1).

**Figure 2:**
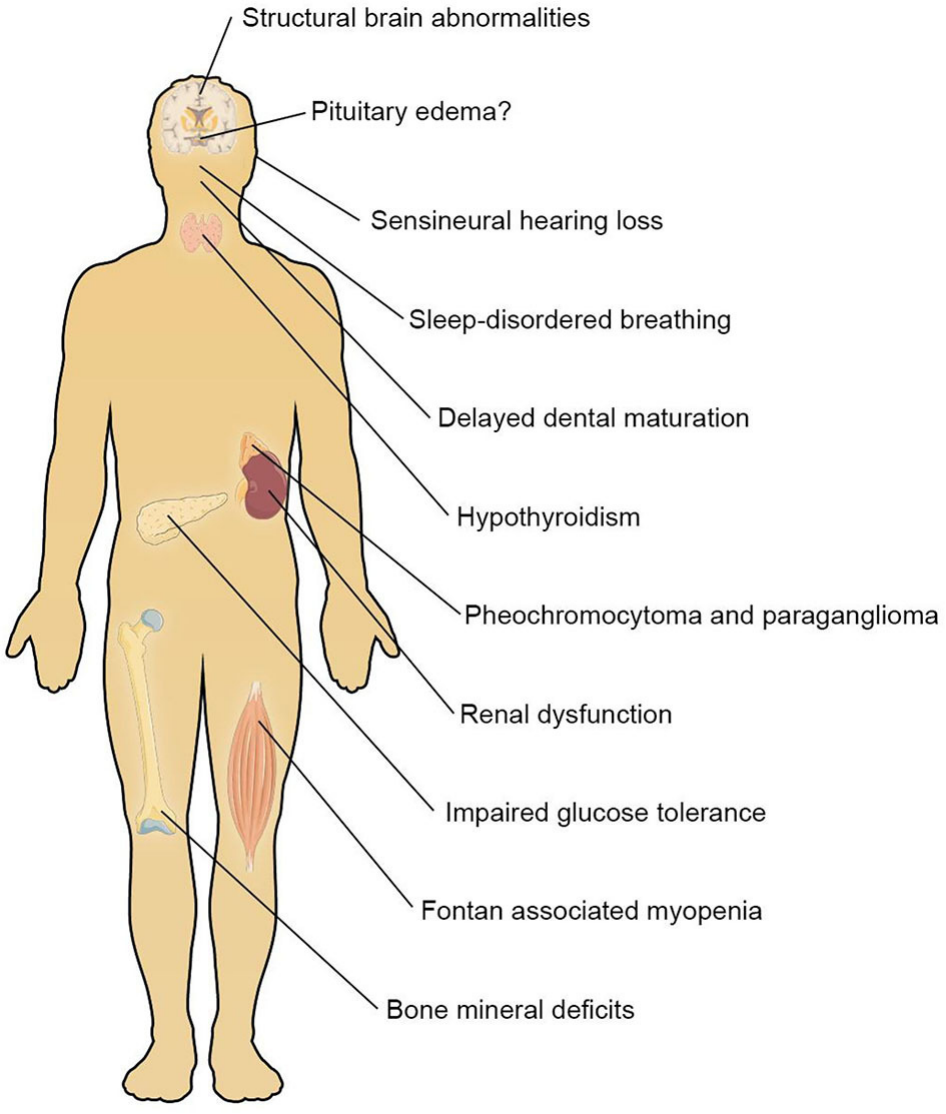
Main findings per organ system.

All included studies are summarized in Supplementary Table 1. The main findings per organ system are summarized in Figure 2.

### Neurology

#### Central Nervous System Abnormalities

Structural abnormalities on central nervous system (CNS) imaging have been assessed in 14 studies using multiple imaging modalities (Table 1). CNS abnormalities noted included (multi)focal abnormalities, developmental malformations, and ischemic changes in watershed areas (9–16). Most of these studies included only adolescent Fontan patients. Verall et al. found adult patients had more impaired neurocognitive scores compared to adolescent patients, however, no relation was found between age and severity of abnormalities on neuro-imaging (16). Diffuse CNS abnormalities in univentricular CHD patients are present even before birth, possibly relating to exposure to hypoxia *in utero* (10, 11). Following each cardiac surgery, ischaemic abnormalities appear to increase in number and size (10). Of note, one study found that in a cohort of 144 adolescents 13% had evidence of stroke on MRI after Fontan completion (10). Remarkably, 40% of these patients did not have a clinical history of stroke, suggesting that the occurrence of stroke can be clinically silent (10). Total and regional brain volumes are generally decreased in Fontan patients compared to controls (17, 19–22). All studies assessing brain volumes included only adolescent patients and no studies assessed brain volumes in adult patients. Decreased brain volumes may relate to prolonged cyanosis in infancy, decreased cardiac output, nutritional deficiencies, and peri-operative injuries (11, 19). Whether these findings relate to neurocognitive outcome is not clearly established. Contrary to other brain structures, the pituitary has an increased volume in Fontan patients (18). This is further explored in the *Endocrinology* section of this review. Fontan patients underperform compared to their peers on several functional measures, including visual processing speed, psychomotor function, emotional cognition, and gross motor function (10, 23, 24). Imaging abnormalities related to functional outcomes in some (9, 13, 14), but not all studies (10, 16).

**Table 1:** Neurologic imaging abnormalities.

**References**	**Patient population**	**N**	**Controls**	**Imaging techniques**	**Main findings**	**Relation with functional outcomes**	**Comments**
**Structural abnormalities**							
Sarajuuri et al. (9)	U-CHD patients median age 5.7 years (range 5.0–7.5)	27	N/a	1.5T MRI. CT when contra-indications	Ischaemic changes in watershed areas in 7/20 (35%) patients	Full-scale IQ lower in group with abnormalities (97 vs. 69, *p* = 0.045)	
Bellinger et al. (10)	Fontan patients aged 15 ± 3 years	144	111 healthy controls	1.5T MRI or 3T MRI incl T2 weighted acquisition	Any abnormalities in 66% of patients. Mostly focal or multifocal abnormalities	Focal abnormalities related to several behavioral disorders, but not general intelligence	*
Watson et al. (11)	Fontan patients aged 15 ± 3 years	128	48 healthy controls	1.5T MRI or 3T MRI incl. cortical thickness	Any abnormalities in 65% of patients	Not assessed	Study assessed both structural abnormalities and regional volumes*
Pike et al. (12)	Fontan patients aged 16 ± 1	20	36 healthy controls	T2-relaxometry MRI	Widespread higher T2-relaxation values	Not assessed	**
Watson et al. (13)	Fontan patients aged 15 ± 3	102	47 healthy controls	Diffusion tensor imaging	Evidence of widespread altered white matter microstructure	Fractional anisotropy for several tracts correlated with both full scale IQ and processing speed	*
Singh et al. (14)	Fontan patients aged 16 ± 1	18	31 healthy controls	3T MRI	Decreased gray matter density in several regions	Prefrontal, occipital, and temporal gray matter density relates to mood and cognitive ability	**
Singh et al. (15)	Fontan patients aged 16 ± 1	27	35 healthy controls	Diffusion tensor imaging	Multiple brain sites in U-CHD showed increased MD values	Not assessed	**
Verrall et al. (16)	Fontan patients aged 23 ± 8	100	41 healthy controls; 50 TGA patients	3T MRI incl T2 weighted acquisition	Structural brain injury in 100% of subjects	Only white matter injury was associated with worse paired associate learning. Severity of infarct, subcortical gray matter injury and microhemorrhage unassociated	
**(Regional) volumes**							
Watanabe et al. (17)	HLHS patients subset of cohort aged 16 ± 6 y	4	19 healthy controls	1.5T MRI	Decreased frontal gray matter volume in HLHS patients	Not assessed	***
Watson et al. (11)	Fontan patients aged 15 ± 3 years	128	48 healthy controls	1.5T MRI or 3T MRI incl. cortical thickness	Reduced volumes in 29% of regions. Cortical thickness reduced in 50% of regions	Not assessed	Study assessed both structural abnormalities and regional volumes
Muneuchi et al. (18)	Fontan patients aged 9 [8–12]	40	40 healthy controls	1.5T MRI	Increased pituitary volumes	Not assessed	***
Cabrera-Mino et al. (19)	Fontan patients aged 16 [15–17]	25	38 healthy controls	3T MRI	Reduced mammilary body volume	Corrected mamillary bodies volumes correlated with MoCA and delayed memory recall scores in SVHD and controls	**
Noorani et al. (20)	Fontan patients aged 16 ± 1	23	37 healthy controls	3T MRI incl T2 weighted acquisition	Significantly reduced caudate volume	Caudate volumes correlated with PHQ-9, BAI, GMI, and MoCA scores	**
Hiraiwa et al. (21)	Fontan patients aged 9 ± 2	18	9 TGA patients	1.5T MRI	Smaller TBV compared to TGA patients	Full-scale IQ correlated with total brain volume (9 TGA patients included)	Only last follow-up considered, as not all patients had undergone Fontan palliation at first visit***
Pike et al. (22)	Fontan patients aged 16 ± 1	25	38 healthy controls	3T MRI	Reduced right, but not left, hippocampal volume	WRAML2 scores correlated with hippocampal volumes	**

U-CHD, univentricular congenital heart disease; HLHS, hypoplastic left heart syndrome; TGA, transposition of the great arteries; MRI, magnetic resonance imaging; CT, computed tomography; TBV, total brain volume; MoCA, Montreal cognitive assessment; PHQ, patient health questionnaire; BAI, Beck anxiety inventory; GMI, Global mindset inventory; WRAML2, Wide range assessment of memory and learning 2. *, **, and *** denote studies with overlapping study populations.

#### Cerebral Hemodynamics

Cerebral hemodynamics have been assessed by 3 studies (25–27). Saiki et al. investigated the response to inferior caval vein occlusion during cardiac catheterization (to isolate the upper body circulation as a proxy for cerebral circulation) in Fontan patients aged 6.7 ± 2.6 years (25). They found Fontan patients have an increased cerebral-to-systemic cardiac output ratio compared to patients with structurally normal hearts. This might reflect a compensatory mechanism to preserve cerebral blood flow in a context of reduced cardiac output. Cerebral local tissue oxygenation, assessed by near infrared spectroscopy, decreases during exercise for pediatric Fontan patients (26). This response is not seen for healthy controls. Cerebral deoxygenation during exercise may play a role in the impaired exercise performance of Fontan patients. Wong et al. found adult Fontan patients have an impaired response in cerebral blood flow to cognitive stimuli compared to controls (27).

#### The Autonomic Nervous System

Adult Fontan patients have increased circulating levels of norepinephrine (28, 29), increased sympathetic tone of muscle nerves (30), and reduced heart rate variability (31, 32). Each of these factors is considered a marker of increased autonomic sympathetic tone. The age of patients in these studies ranged from the early twenties to late thirties. One study evaluated the autonomic nervous system in pediatric Fontan patients (33). Similar to the findings in adults, the authors found increased circulating levels of norepinephrine and reduced heart rate variability (among other factors relating to sympathetic tone). Increased sympathetic tone can be a compensatory mechanism in heart failure (30). Heart rate variability may be explained by other factors than increased sympathetic tone in Fontan patients. Indeed, heart rate recovery following maximal cardiopulmonary exercise testing—a process also governed by the autonomic nervous system- was normal in Fontan patients (31). Ohuchi et al. found sympathetic activity was not related to hemodynamics, clinical history, or time since Fontan completion (33). Sympathetic activity seems increased for Fontan patients from childhood, although the clinical consequences—including the effects on heart rate and clinical prognosis-remain uncertain.

#### Central Nervous System Infections

Our search returned 4 case reports of Fontan patients (aged 11–28 years) with cerebral abscesses (34–37). Fontan patients are considered to be at increased risk for hematogenous spread of pathogens to the central nervous system due to shunting (37). In a nationwide cohort study in Denmark the risk of CNS infections (not limited to cerebral abscesses) was 0.93 per 1,000 person years for univentricular CHD patients (38). This represents a 3-fold (1.13–8.17) increased risk compared to the general population.

#### Headaches

In a cross-sectional study of 54 Fontan patients, aged 26 ± 9 years, 50% of patients complained of frequent headaches (39). We found one case report of a Fontan patient with frequent headaches caused by pseudotumor cerebri (40). Pseudotumor cerebri is a condition caused by elevated intracranial pressure, which may relate to Fontan physiology. More research is needed to estimate the prevalence of pseudotumor cerebri in Fontan patients, but pseudotumor cerebri may be considered as a cause for refractory headaches in Fontan patients.

### Renal System

Renal dysfunction (RD) has been demonstrated in Fontan patients in 8 studies (41–50). The overall prevalence of RD (defined as estimated glomerular filtration rate (eGFR) <90 mL/min/1.73m^2^, as assessed by creatinine-based methods) in Fontan patients is estimated to be 10–20% (41–43, 49). Moderate to severe RD (eGFR_creatinine_ <60 mL/min/1.73m^2^) was found in 1–4% of Fontan patients (41–43, 49). Renal dysfunction was generally less common in cohorts of younger patients (41, 44, 48), compared to those with older patients (42, 43, 50).

The use of creatinine-based GFR estimation methods have been debated, since muscle mass is often reduced in Fontan patients. Alternative methods may be more reliable (42–44, 51). Cystatin C is a marker of renal function that is not dependent on muscle mass (44). Differences between eGFR_creatinine_ and eGFR_cysC_ can be large in the Fontan population (42–44, 51). There is no consensus yet on the appropriate use of eGFR_cysC_ or eGFR_creatinine_ in Fontan patients. No studies compared cystatin C GFR estimations to invasive measurements. Further studies are necessary to determine the best method to non-invasively evaluate renal function.

#### Pathophysiology

The pathophysiology of renal dysfunction in Fontan patients is incompletely understood. Cyanosis, erythrocytosis, limited perfusion, and renal congestion have been suggested as factors leading to renal dysfunction (48, 49, 52–55). The histopathological features of the kidneys of Fontan patients with renal dysfunction range from mild changes to focal segmental glomerulosclerosis (55–57). Adaptive focal segmental glomerulosclerosis is a renal disease commonly seen in patients with cyanotic CHD. In contrast, we also found one case report where the structures of tubules and glomerulus were preserved despite severe renal dysfunction (55). In this case renal dysfunction may be reversible (55). Focal segmental glomerulosclerosis may occur in older patients (ages of case reports ranged from 24 to 41 years old) (56, 57) compared to renal dysfunction with preserved renal structure (one case report in a 14 year old patient) (55).

#### Longitudinal Assessment

Four studies assessed renal function longitudinally (41, 47, 51, 58). Overall, most studies find renal function decreases over time for Fontan patients (47, 51, 58). In one cohort study creatinine increased during a 5-year follow-up (58). In a cross-sectional study the rate of decline in invasively measured GFR was 9.92 ml/min/1.72m^2^ per decade following the Fontan procedure (51). Motoki et al. found a significantly higher eGFR_creatinine_ in adolescent Fontan patients vs. the adult Fontan patients (113 ± 25 vs. 147 ± 19 ml/min/1.72m^2^, *p* < 0.01) (47). Only the study by Khuong et al. found no overall decrease in renal function over a 7 ± 5 year follow-up (41). Nevertheless, a subgroup analysis showed that patients with normal renal function at baseline had decreasing renal function over the follow-up duration (41).

#### Prognostic Value of Renal Function

Several studies assessed the prognostic value of renal function for clinical outcomes, such as hospitalization and death for Fontan patients. Cystatin C predicted a composite endpoint of non-elective cardiovascular hospitalization and mortality, adjusted for age and NYHA class [HR for RD 3.25 (95% CI 1.26–8.40)] (43). Creatinine based GFR predicted mortality in some (41, 50), but not all studies (43, 46). One study found eGFR_creatinine_ predicted unscheduled hospitalization (46). Ohuchi et al. (46) found creatinine predicted unscheduled hospitalization for adults, but not children. Overall, renal function seems to relate to clinically important long term outcomes.

#### Other Findings

Proteinuria is a marker of kidney damage. Five studies assessed proteinuria in Fontan patients and found a prevalence of 10–65% (42, 44, 51, 52, 54). One study found the prevalence is 3 times higher compared to the general population (52). The presence of proteinuria does not seem to be related to GFR, implying these phenomena may have different underlying processes (44). Renal vascular resistance is higher for Fontan patients, and correlates with renal function, exercise capacity, and mortality during a median 32 months follow-up (59). In a study of structural abnormalities of the kidneys several findings were reported: increased parenchymal echogenicity in 6%, cortical thinning in 4%, discrete scarring in 4%, pelvicalyceal system dilatation in 1%, and enlargement of the kidneys in 3% of patients (51).

### Muscular System

Our search returned eighteen articles discussing the muscular system in Fontan patients. Lean muscle mass is decreased for Fontan patients compared to healthy controls (60–63). Fontan associated myopenia, defined as a lean muscle mass Z-score of −2 or lower, is present in 39% of Fontan patients (63). Bio-impedance measurements and MRI-derived muscle mass estimations—which are more easily obtained estimations of muscle mass- are also abnormal for Fontan patients (64–66). No studies directly compared these methods of lean muscle mass estimation to current gold standards. Nevertheless, these methods may provide more easily obtainable measurements in clinical practice.

#### Factors Related to Muscle Mass

Lean muscle mass Z-scores were not related to age for Fontan patients (62). However, isometric knee extension muscle strength is impaired for adolescent Fontan patients, but not during childhood, which suggests that muscular impairment increases with age (67). Markers of neurohormonal activation, sex hormones, and inflammatory mediators did not relate to lean muscle mass, implying the role of these factors is limited in the pathogenesis in myopenia (63). Vitamin D deficiency is common in Fontan patients (62, 63). Studies into the effect of vitamin D status on muscle mass found conflicting results (62, 63). Furthermore, Fontan patients have less active lifestyles than healthy peers, which may play a role in muscle mass development (68). Overall, determinants of lean muscle mass deficits in Fontan patients are not clearly established. Few hypotheses regarding the pathophysiology of Fontan associated myopenia have been proposed.

#### Muscle Mass Related Functional Outcomes

Fontan patients have impaired exercise capacity, as well as decreased functional parameters of muscle strength (69–71). The role of lean muscle mass on exercise capacity in Fontan patients is uncertain (63, 65). The pathophysiology of exercise impairment in Fontan patients is likely multi-factorial. It has been hypothesized that the calf muscle pump plays an important role in augmenting single ventricular preload in the context of a Fontan circulation. Calf muscle size is reduced for Fontan patients compared to healthy references (68). Leg lean mass relates to exercise capacity as well as cardiac index during exercise (72). Importantly, Cordina et al. found that peak oxygen uptake, muscle strength and total muscle mass could be increased by resistance training (73). Resistance training could be a valuable treatment modality for cardiovascular, and general, health in Fontan patients.

#### Muscle Oxygenation

Six articles investigated the oxygenation of muscles in Fontan patients (26, 61, 74–76). Several studies evaluated muscle oxygenation with near-infrared spectroscopy (26, 74, 76). Muscle oxygenation is decreased at baseline, relating to reduced arterial oxygen saturation, but displays a normal response to exercise (26). Several markers of muscle oxygenation during exercise—but not resting conditions- relate to overall exercise capacity (74). Muscle oxygenation defects may increase with age (76). Parameters of muscle oxygenation during exercise may be preserved in children (6–12 years), but impaired for adolescents (13–18 years) (76). Vandekerckhove et al. did find impaired muscle oxygenation during exercise in patients aged 11.8 ± 2.8 years (26).

Studies evaluating muscle energy metabolism by phosphate spectroscopy found phosphocreatine recovery rate, a measure of aerobic capacity, was reduced in Fontan patients aged 30 ± 2 years compared to controls (61). Fore-arm blood flow, which is considered a proxy for general muscular blood supply, relates to reduced skeletal muscle diameter and strength in Fontan patients (75). Decreased muscular oxygenation may be an important determinant of exercise capacity in Fontan patients.

### Endocrinology

The pituitary is an important organ in many endocrine axes. The volume of the pituitary, in contrast to most brain structures, is larger in Fontan patients than control subjects (18). Pituitary volumes in Fontan patients increased with age, but at a rate similar to healthy controls (18). Increased pituitary volumes may relate to the circulation of the pituitary, which has a portal system, similar to the liver. The large pituitary volumes may reflect pituitary edema, which may have consequences for hormone production. Several endocrine axes are discussed below. No studies assessed the growth hormone axis in Fontan patients.

#### Thyroid Axis

Three studies investigated the thyroid axis (39, 50, 77). Kuwata et al. in a retrospective study of 35 pediatric Fontan patients who underwent cardiac catheterization, found 12 patients (33%) had subclinical hypothyroidism (77). In retrospective cohort studies 13–55% of Fontan patients were diagnosed with hypothyroidism at follow-up (39, 50). TSH levels related to ventricular function and central venous pressure, implying Fontan physiology (including pituitary congestion) may play a central role in hypothyroidism (77). Amiodarone therapy has been proposed as a cause for hypothyroidism in Fontan patients. However, no patients in the previously mentioned study by Kuwata et al. used amiodarone (77). It should be noted no studies prospectively studied the thyroid axis at long-term follow-up for Fontan patients. How thyroid function develops with a long-standing Fontan circulation is currently not known.

#### Parathyroid Axis

Sharma et al. found, in a retrospective study of 68 pediatric Fontan patients, that parathyroid hormone levels were highly increased in Fontan patients compared to healthy controls [59 (43–59, 61–82) pg/mL vs. 23 (17–30) pg/mL, *p* < 0.001] (44). Other studies in children found comparable parathyroid hormone levels for Fontan patients (78, 79). No studies assessed parathyroid function in adults. Parathyroid hormone levels do not relate to exercise capacity (78).

Serum parathyroid levels are mainly regulated by serum calcium status, but also influenced by—among others- phosphate status, renal function, and vitamin D sufficiency. Two studies found a high prevalence (70 and 81%, respectively), of vitamin D deficiency in Fontan patients (78, 79). Vitamin D supplementation can increase vitamin D levels and decrease parathyroid levels (49 ± 32 vs. 68 ± 41 ng/L, *p* < 0.001) (79). Due to discrepancies between several studies, the exact etiology and clinical importance of hyperparathyroidism in Fontan patients remains unclear. Nevertheless, it seems important to monitor vitamin D levels on a regular basis for these patients.

#### Renin-Angiotensin-Aldosterone System

Fontan patients have increased serum renin, angiotensin II, and aldosterone levels compared to healthy controls in both children (80) and adults (81). Plasma sodium levels (Na) are heavily affected by the renin-angiotensin-aldosterone system and diuretic use. One study found 30% of Fontan patients have hyponatremia (82). Plasma Na increased with age for patients without medication use (82). Plasma Na is an independent predictor for unscheduled rehospitalization (82). Another study found a correlation between plasma renin activity and renal vascular resistance (59). Abnormalities in the renin-angiotensin-aldosterone axis are well-described. However, the clinical value of these measurements seems limited and are heavily confounded by medication use.

#### Sex Hormones

Menon et al. in a cross-sectional study of 299 adolescent Fontan patients, assessed Tanner stages (using a self-assessment pictorial Tanner stage questionnaire), and found that more than half of the Fontan patients (58%) had a delay in one of the Tanner stage parameters (83). There was a median delay of 1.5–2 years between Fontan patients and the normal population in achieving the Tanner stages in both sexes. The only independent factor associated with the delay in puberty was a history of more than two cardiac surgeries. Besides multiple surgeries with cardiopulmonary bypass, delayed puberty is thought to be caused by decreased cardiac output, cyanosis, and endocrine abnormalities (83).

#### Apetite-Related Hormones

Shiina et al. in a prospective study of 46 adult Fontan patients, showed that in the Fontan patient group plasma ghrelin levels, a hormone primarily related to increased appetite, were lower than those in controls (*P* < 0.05) (66). Ghrelin is an orexigenic hormone. It has many functions, including regulation of glucose and fat metabolism and stimulation of GH release. Furthermore, it has a favorable effect on cardiovascular function (decreases vascular resistance and increases cardiac output). It is seen as a potential therapeutic target in congestive heart failure (66). In Fontan patients, the decreased ghrelin levels may contribute to cardiovascular dysfunction, growth stunting and metabolic abnormalities. However, the exact role of ghrelin in these processes remains poorly understood.

### Metabolism

#### Glucose Metabolism

Studies into the glucose metabolism have elucidated several abnormalities: adult Fontan patients have lower fasting glucose (29, 84). However, HbA1c -a marker of long term glycemic status- and C-peptide—a marker of endogenous insulin production- are both increased (29). The results from an oral glucose tolerance test are more unfavorable for Fontan patients compared to healthy controls (29, 85). A large study of 275 patients found thirty-four percent of patients had impaired glucose tolerance and five percent had diabetes mellitus (85). Furthermore, oral glucose tolerance decreased over a follow-up of 6.5 ± 2.7 years for a subset of 175 patients aged 20 ± 7 years (85). Remarkably, HbA1c decreased during this time frame (85). Fasting glucose is increased in pediatric patients, although glucose tolerance may be preserved at this age (in contrast to in adults) (84, 85).

The abnormal glucose metabolism may be related to hepatic dysfunction in Fontan patients, as the liver plays an important role in glucose homeostasis (85). The (paradoxically) decreased HbA1c may relate to an increase erythrocyte turnover due to residual hypoxia in Fontan patients (85). Furthermore, myopenia may contribute to plasma glucose levels, as skeletal muscle is a major consumer of plasma glucose. Adiponectin, a regulator of glucose metabolism and insulin sensitivity -among other functions-, was higher for Fontan patients compared to controls. The exact pathophysiology of abnormal glucose metabolism in Fontan patients is incompletely understood. Abnormal glucose metabolism was a predictor of overall mortality and unplanned hospitalization in adult patients (46, 85). Furthermore, it related to increased renal vascular resistance (59).

#### Lipid Metabolism

Most (84, 86), but not all (29, 46), studies found Fontan patients have lower total cholesterol compared to healthy peers (84, 86). High density lipoprotein (HDL), non-HDL and low density lipoprotein (LDL) cholesterol subtypes were each decreased (29, 84, 86). Total cholesterol did not differ between pediatric and adult Fontan patients (46). Total cholesterol levels did not predict unplanned hospitalization or mortality (46). The prognostic value of individual cholesterol subtypes was not considered in this study (46). Michel et al. performed a comprehensive metabolic analysis of phospholipid and acetylcarnitine metabolism and found metabolites of each class of lipid (phosphatidylcholine, lysophosphatidylcholine, sphingomyelin, and acylcarnitines) were decreased in Fontan patients (87). In a different metabolomics study including patients with heart failure, similar differences in acylcarnitines between patients and controls were found, and several acylcarnitines and amino acids differed between patients with and without heart failure (88). Saraf et al. found adiponectin, a hormone of lipid catabolism, was increased in Fontan patients compared to controls (81). During stress, cardiomyocytes may preferentially utilize more glucose, rather than lipid. As such biomarkers of energy metabolism may relate to a heart failure phenotype, which may be used as biomarkers for prognosis.

#### Amino Acid Metabolism

The amino acid metabolism has been studied in Fontan patients with a dominant left ventricle (89). Serum concentration of several amino acids, among which glutamic acid and hydroxyproline, are increased, whereas other amino acids are decreased (among others taurine, asparagine, and threonine). The methionine sulfoxide to methionine ratio is decreased in Fontan patients compared to healthy controls and negatively correlates with exercise capacity (89). A shift in amino acid metabolism may relate to altered myocardial energy metabolism, increased protein turnover, or oxidative stress and endothelial dysfunction (89).

### Neoplastic Disease

Our search returned 6 articles related to neoplastic disease. Diller et al. studied the causes of death for different CHD diagnoses. They found neoplastic disease accounted for 3% of Fontan patients deaths (90). This was lower than the percentage for all CHD patients (6.3%), but noteworthy, higher absolute mortality rates in Fontan patients complicate direct comparisons (standardized mortality ratio was 23.4 for Fontan patients and 2.3 for all CHD patients, both compared to healthy references) (90). Heart failure was the most common cause of death for Fontan patients, accounting for 52% of deaths (90).

Furthermore, our search returned 6 case reports and case series detailing in total 13 Fontan patients—aged 11 to 38.5 years—with pheochromocytoma (PHEO) or paraganglioma (PGL) (91–96). Both are catecholamines-producing neuro-endocrine tumors. Song et al. found a cumulative incidence of PHEO and PGL of 2.5% in Fontan patients >10 years old on active follow-up (91). The risk of PHEO or PGL in patients with cyanotic CHD is increased compared to people without CHD (OR 6.0, 95% confidence interval 2.6–13.7, *p* < 0.0001) (97). Of note, eight out of eighteen of the cyanotic CHD patients in this study had a Fontan palliation (97).

Two cases of neuroendocrine tumors (NET) were described in a case series by Vural et al. (98). A grade 2 NET of the jejunum was found in a 20 year old woman and a pancreatic NET was found in a 12 year old boy (98).

Chronic hypoxia could play an important role in the development of neoplasms (91–96). Hypoxia inducible factors (HIFs) have been proposed to induce tumor growth in PHEO and PGL (92). Furthermore, underlying genetic defects may predispose to both univentricular CHD and neoplasma (91, 94). Clinicians should be aware of the higher risk of PHEO and PGL in Fontan patients and be vigilant for clinical symptoms of excess catecholamine production.

### Bone Abnormalities

Our search on bone abnormalities in Fontan patients yielded 9 articles (39, 68, 78, 99–104). Five studies assessed bone mineral density (BMD) Z-scores measured by either Dual Energy X-ray Absorptiometry (DEXA) or peripheral Quantitative Computed Tomography (pQCT). Each found BMD is mildly decreased in Fontan patients compared to age-related references (68, 99, 100, 102, 104). These studies are summarized in Table 2. BMD Z-scores are within normal range for the majority of patients. D'Ambrosio et al. found a prevalence of 29% for a BMD in the osteopenic range (i.e., Z ≤ 1) and 4% (*n* = 1) in the osteoporotic range (Z ≤ 2.5) (99). Diab et al. found a Z-score of −2 in 20% (*n* = 13) of their study population (100). The prevalence was lowest in the youngest age group (5–9 years, 5%) and highest in oldest age group (16–18 years, 35%). This is worrisome, as most studied Fontan patients are relatively young, and may develop osteoporosis in adulthood. When indexing BMD values to references (such as Z-scores) the delayed development of children with a Fontan circulation should be taken into account. Since Z-scores are age dependent, a delay in puberty can lead to a deceptively low BMD. Bone age can be determined by hand radiograph, and bone-age-related Z-scores, rather than calendar age-related Z-scores, may provide a more precise way of indexing BMD for Fontan patients (100).

**Table 2:** Bone mineral density Z-scores.

**Study**	***N***	**Age (years)**	**Measurement**	**BMD Z-scores**		***P*-value**
				**Location**	**Value**	
Witzel et al. (104)	6	18.6 ± 3.1	pQCT	Radius trabecular	0.0 ± 1.0	0.98
Sarafoglou et al. (102)	10	12.1 ± 1.8	DEXA	L1–L4	−0.5 ± 1.1	Not reported
				Total body	−0.6 ± 1.1	Not reported
			pQCT	Radius trabecular	−30 (95% CI: −59, −1)*	**0.041**
				Radius cortical	−15 (95% CI: −64, 35)*	0.533
				Radius total	−42 (95% CI: −87, 4)*	0.070
Diab et al. (100)	64	5–18	DEXA	L2–L4	−1.0 ± 1.3	Not reported
				Total body	−0.2 ± 1.2	Not reported
D'Ambrosio et al. (99)	28	26 ± 7	DEXA	Hip	−0.6 ± 1.1	**0.01**
				Spine	−0.7 ± 1.1	**<0.01**
Avitabile et al. (68)	43	12.8 [5.1–33.5]	pQCT	Tibia trabecular	−0.9 ± 1.0	**<0.001**
				Tibia cortical	−0.2 ± 1.0	0.27

pQCT, Peripheral quantitative computed tomography; DEXA, dual energy X-ray absorptiometry; CI, confidence interval; ^*^Raw data provided instead of Z-scores. P values < 0.05 were considered statistically significant and are shown in bold.

#### Pathophysiology

Several hypotheses for the pathophysiology of BMD deficits in Fontan patients have been proposed. Hypoxia and reduced cardiac output are common features of Fontan physiology. Hypoxia may increase osteoclast activity and bone resorption (39, 99). Reduced circulation of bone marrow, in the context of reduced cardiac output, may impair bone formation (103). Disturbances in the calcium-vitamin D-parathyroid hormone axis have been reported in Fontan patients (68, 99, 100, 102). The use of some medications, such as glucocorticoids, may have an impact on bone health (68, 100). Physical activity is a necessary stimulus for optimal bone development (68). Fontan patients may have less active lifestyles compared to healthy peers (68). Sarafoglou et al. found weight bearing bones were less affected than non-weight bearing bones, which may relate to physical exercise (102).

#### Serum Biomarkers of Bone Health

Serum biomarkers related to bone development have been studied in Fontan patients. Bone Specific Alkaline Phosphatase (BALP) serum levels, a biomarker produced by osteoblasts, are reduced in Fontan patients compared to controls (103). Osteopontin, a component of the bone matrix, is decreased in the serum of Fontan patients compared to controls (102). No studies directly related BALP or osteopontin to BMD or clinical outcomes. The clinical implications of these biomarkers in Fontan patients are currently unclear, but they might represent a biomarker of bone metabolism that is easily obtained in clinical practice (103).

#### Clinical Outcomes

The risk of fractures related to osteoporosis has not extensively been studied in Fontan patients. One study described the occurrence of fractures and found no patients with a history of frequent fractures (100). Acquired scoliosis is more common in Fontan patients compared to the general population (12 vs. 2–3%) (101). Furthermore, the prevalence of scoliosis increased with age (estimated prevalence 0–10% for patients with 0–5 years of follow-up since Fontan completion vs. 30–45% for patients with >10 years of follow-up) (101). Scoliosis is most commonly considered a complication of (often multiple) thoracotomies related to Fontan surgery, but decreased BMD may also play some part in the etiology of scoliosis in Fontan patients (39).

### The Immune System

We found 3 studies which described the prevalence of lymphopenia in Fontan patients (without PLE), and one study describing cytokine expression in Fontan patients (81, 105–107). Rates of lymphopenia (defined as a lymphocyte count below 1,000 cell/ml) ranged from 12 to 32% (105–107). Morsheimer et al. found the proportion of patients with lymphopenia to be significantly higher in patients who had a Fontan palliation over 10 years ago, whereas Alsaied et al. found no difference in age between patients with and without lymphopenia (105, 107). Lymphopenia in Fontan patients may be related to Fontan associated liver disease (105, 106), (subclinical) enteric lymph loss –a similar pathology to PLE-(107), and overall lymphatic congestion due to increased central venous pressure (78). No studies assessed the effects of lymphopenia on risk of infective diseases for Fontan patients.

Cytokine expression has been analyzed by Saraf et al. (81). Different types of pro-inflammatory cytokines were significantly elevated in Fontan patients compared to healthy controls. These included Tumor Necrosis Factor-α, Interleukin-6, Growth/differentiation Factor-15, and β2-macroglobulin. This could indicate that even clinically stable Fontan patients may have chronic subclinical inflammation, similar to patients with congestive heart failure (81).

### The Auditory System

We found 4 studies detailing disorders in the auditory system in Fontan patients. Gopineti et al. estimated the prevalence of sensorineural hearing loss (SNHL) in children who had undergone congenital heart surgery (108). The prevalence in Fontan patients was 14.7% (11 out of 75), compared to 0.35% in the general adolescent population. Other studies found a prevalence of hearing disability in 0–13% of pediatric Fontan patients (9, 23, 24). Overall, we found 14 cases out of 151 study subjects (9%) in these studies (9, 23, 24, 108). The age of patients in these studies ranged from 5 to 12 years. No studies assessed hearing disability in adult patients. Hearing disabilities may be caused by inner ear ischemia, hypo perfusion, hypoxia and high doses of furosemide (108). Audiology screening at regular intervals in childhood for all patients with a Fontan circulation seems reasonable.

### Reproductive System

Our search returned 4 articles in total, of which 3 articles regarding abnormalities in the female sex organs (109–111) and one regarding sexual function in males (112).

Two studies assessed the placenta in pregnant patients with a Fontan circulation (109, 110). In total 31 pregnancies were described, of which 12 pregnancies resulted in live births (109, 110). Only 7 pregnancies were delivered at term (109, 110). Both studies found placental weight varied significantly across pregnancy. An important proportion of placentas had low weight for gestational age (109, 110). Histological examination revealed all placentas had some form of histological hypoxic lesions. Philips et al. found prominent sub-chorionic fibrin deposition in all placentas (110). In the study by Yokouch-Konishi et al. (109) seven of eight placentas had a chronic subchorionic hematoma. Other histopathologic changes included lesions associated with maternal vascular malperfusion, villous stromal fibrosis, and placental hypervascularity. The authors speculate that Fontan physiology may be associated with these placental abnormalities. Chronic hypoxemia, high systemic venous pressures and low cardiac output may play a part in the development of poor placental health, and subsequently pregnancy outcomes, in Fontan patients (110).

Canobbio et al. found most (69%) female Fontan patients have normal menstrual patterns (111). Some (31%) Fontan patients older than 18 years reported abnormal flow patterns during menstruation. These complaints included oligo-menorrhea, amenorrhea, and menorrhagia. No comparison to healthy peers was made in this study. The authors propose prolonged cyanosis may relate to menstrual disorders. It should be noted subjects in this study underwent Fontan completion at an old age (mean 18.3 years), compared to current clinical practice (111). Extrapolating these findings to contemporary Fontan patients may therefore be inappropriate.

Rubenis et al. in a survey of 54 male Fontan patients aged 28 ± 3 years, studied sexual function in Fontan patients (112). The prevalence of erectile dysfunction did not differ between patients and controls. Overall satisfaction was lower in Fontan patients (8.3 ± 1.9 vs. 9.5 ± 0.8 out of 10, *p* < 0.001), relating to reduced sexual desire. Other domains of sexual satisfaction did not differ vs. controls. It should be noted only 40% of eligible patients participated in this survey. Due to the sensitive nature of the survey, selection bias and self-reporting may have confounded the results of the study. Chronic venous congestion and decreased perfusion are considered risk factors for erectile dysfunction (112). Health care providers could address concerns regarding sexual health for Fontan patients.

### Sleep-Disordered Breathing

Our search yielded three articles related to sleep disordered breathing in Fontan patients (113–115). Sleep-disordered breathing, which includes obstructive sleep apnea (OSA), is often seen in patients with acquired heart failure, but its role in congenital heart disease is less extensively studied (113). One retrospective study found twenty-two out of fifty-five (40%) Fontan patients underwent polysomnography (PSG) at any time (113). Out of these patients 77% was diagnosed with sleep-disordered breathing (31% of the total study cohort). Forty one percentage suffered from nocturnal hypoxemia (absolute desaturation of ≥5% from baseline saturation) and 36% suffered from OSA. Sleep-disordered breathing incidence or severity did not relate to patients' age. OSA can cause hemodynamic changes due to obstruction of the upper airway, hypoxic pulmonary vasoconstriction, and increased pulmonary vascular resistance. This could be a substantial problem in Fontan patients, where blood flow to the lungs is entirely passive (113). Health care providers should be alert for symptoms of sleep-disordered breathing-such as daytime somnolence, fatigue, and cognitive decline- in Fontan patients, which may easily be confused for general symptoms associated with a Fontan circulation.

Continuous positive airway pressure (CPAP) ventilation, the gold standard for OSA treatment, may have detrimental effects on the Fontan circulation by increasing intrathoracic and pulmonary arterial pressure (114). Echocardiography or cardiac catheterization can be used to titrate CPAP parameters and optimize airway pressures with regard for circulatory function (114, 115).

### Dermatology

Our search returned 3 articles, of which 2 regarding lower limb varices and one regarding impaired wound healing. Varicose veins and venous insufficiency of the lower extremities are common features in the general population. Bhatt et al. found no differences in the prevalence of clinical signs of chronic venous insufficiency -such as reticular veins, edema, varicose veins, or ulceration- between Fontan patients and healthy controls (116). However, the prevalence of venous reflux assessed by duplex ultrasonography in Fontan patients was higher compared to healthy controls (51 and 10%, respectively) (116). The prevalence of venous reflux did not relate to patients' age. In a study by Pike et al. 11/54 Fontan patients (20%) were diagnosed with varicose veins of the lower extremities (39). The prevalence of varicose veins, or venous insufficiency, of the lower legs does not seem to be increased in Fontan patients. The calf muscle pump may play an important role in the Fontan circulation by augmenting venous return.

Kovacevic et al. described a case report of a 2-year-old boy with impaired wound healing after—otherwise uncomplicated- neurosurgery for head trauma (117). The impaired wound healing could be related to Fontan physiology as the central venous pressure is coupled with the intracranial pressure. Increased intracranial pressure may thus impair scalp perfusion (117).

### Ophthalmology

Only 2 articles were found detailing the long-term consequences of the Fontan circulation on the eyes or vision. Hagemo et al. found 4 out of 15 HLHS patients who underwent extensive examination at long term follow-up presented with strabismus (23). Another study found vision impairment in 1 of 34 (3%) Fontan patients (24). These were secondary end-points for smaller studies and are not extensively discussed in their respective studies. As such, the prevalence and impact of eye conditions and vision impairment in Fontan patients remains unclear. Early detection of visual deficiencies may provide Fontan patients with adequate support and limit developmental delay from visual impairments. Screening of vision at regular intervals during childhood seems reasonable.

### Dental Disorders

We found two studies concerning dental health in Fontan patients. One study compared 268 healthy controls (age 9.4 ± 3.4 years) to 165 children with CHD (8.0 ± 3.2 years) and 103 children with acquired heart disease (11.4 ± 2.7) years (118). The amount of debris of calculus found on teeth surfaces did not differ between these groups. However, Fontan had lower dental ages (1.1 ± 0.8 years below chronological age). Children with acquired heart disease did not differ significantly from the healthy controls (118). No mechanism for the delayed dental maturation was proposed by authors (118).

As previously discussed, Fontan patients are at increased risk of hematogenous spread of infection due to shunting. We found one case report on a Fontan patient with a brain abscess due to *Strepcoccus gordonii*, a pathogen commonly found in dental plaque (37). This highlights the need for good dental hygiene (37). Good dental health may prevent serious sequelae from bacteraemia in Fontan patients.

### Gastro-Intestinal System

Fontan associated liver disease and PLE are common sequelae of a Fontan circulation, which have been discussed in previous reviews (6, 119). These topics were considered out of the scope of this current review.

One study assessed the hemodynamic response to food ingestion of 15 Fontan patients, aged 27.6 [21.8–34.6] years, compared to 15 healthy controls (120). At baseline Fontan patients have significantly greater baseline regional vascular impedance in the kidneys and legs, but no differences in the superior mesenteric or celiac artery. After food ingestion there are no significant differences in global hemodynamic response and change in systemic vascular impedance, but some regional changes. Most notably, lower limb vascular impedance in healthy controls increases temporarily. However, in Fontan patients lower limb vascular impedance decreases following food intake. The researchers hypothesized the changed responsiveness of the lower leg vascular impedance could be a result of the gut-released vasodilating hormones in response to food ingestion. Fontan patients may not sufficiently be able to counteract this vasodilation by increasing the sympathetic tone in the lower legs (120).

## Discussion

The Fontan circulation impairs systemic circulation, venous return, and lymphatic drainage in all organ systems. The goal of this systematic review was to provide an overview of abnormalities in organ systems beyond the heart, lungs, liver, and gut in patients with a longstanding Fontan circulation. We found abnormalities in many of these organ systems (see Supplementary Table 1). Main findings –those that have been described in various studies- are discussed below, as well as findings which are of concern, but are currently more scarcely studied.

### Main Findings

#### Structural Brain Abnormalities

Structural brain abnormalities are common in Fontan patients (9–11, 13, 14, 16). The volume of several brain structures was decreased (17, 19–22). Whether this relates to functional outcomes remains unclear (17, 19–22). Central nervous system abnormalities are present *in utero* and exacerbate during patients' lifetime, most likely relating to peri-operative injuries and thrombo-embolic events (10, 11). Peri-operative neuroprotection strategies, such as anti-inflammatory drugs or ischaemic preconditioning, may limit peri-operative injury (121, 122). Neurodevelopmental screening programs during childhood need to be performed on regular basis and therapies should be employed if needed and available (123).

#### Bacteremia and CNS Infections

Fontan patients are at 3-fold increased risk of CNS infections compared to healthy peers (38). This may relate to hematogenous spread of pathogens due to shunting. Current guidelines recommend endocarditis prophylaxis in patients with e.g., recent intra-cardiac surgery, cyanosis, prosthetic valves, or prior endocarditis (124, 125). Good dental hygiene may prevent bacteremia (37).

#### Musculoskeletal System

We found abnormalities in the musculoskeletal system in Fontan patients. Both muscle and bone mass are decreased in Fontan patients (61–63, 68, 99, 100, 102, 104). Fontan patients may have bone mineral density defects from a young age. Reduced bone mineral density may relate to cyanosis, reduced cardiac output or reduced physical activity. Fontan patients can safely participate in physical exercise, which may improve muscle mass and improve bone development, among other health benefits (126).

#### Renal Dysfunction

Renal dysfunction is a common problem in the Fontan population. Renal function deteriorates over time, but may be present even in cohorts of pediatric patients, as early as a median 10 years after Fontan completion (44). Renal structure may be preserved despite severe renal dysfunction, in which case renal dysfunction may be reversible (55). The European Society of Cardiology (ESC) guidelines for adults with CHD advise annual renal function assessment, but do not provide advice on the method of renal function assessment (124). Creatinine-based GFR estimations can be confounded by Fontan-associated myopenia (42, 43). Cystatin C can be used to estimate GFR independent of muscle mass (127, 128). However, the accuracy of cystatin C remains to be determined in Fontan patients, as thyroid dysfunction and glucocorticoid activity may affect cystatin C levels (129). We advise screening of renal function with both cystatin C and creatinine. Future research should focus on the optimal method of renal function assessment.

#### End-Organ Findings of Potential Concern

The following findings were less frequently reported in literature but nonetheless of potential concern. The volume of the pituitary was larger in Fontan patients than in control subjects (18). This is in contrast to most brain structures, which are smaller in Fontan patients. The pituitary has a portal circulation (similar to the liver) (18). The pituitary as such may be especially vulnerable to increased central venous pressure, which may lead to pituitary edema and hormonal disturbances. Few studies have systematically assessed pituitary hormone axes in Fontan patients. Small studies found hypothyroidism is a common problem, with an estimated prevalence between 13 and 55% (39, 50, 77). Other hormone axes have scarcely been studied. Studies regarding the growth hormone axis would be of particular interest, given growth stunting and delayed puberty—processes influenced by growth hormone axis activity—occurs in Fontan patients and the growth hormone axis is usually the first affected axis in panhypopituitarism (130).

With regard to malignancies in Fontan patients, we found 13 case reports of neuroendocrine tumors, especially pheochromocytoma and paraganglioma (91–96). Chronic hypoxia or underlying genetic defects—which may also underlie the congenital heart defect—could play an important role in the development of such neoplasms (91–96). Health care providers should be aware of the risk of NET in Fontan patients and be vigilant for clinical symptoms of excess catecholamine production.

Hearing loss -although reported only in smaller retrospective studies- was remarkably prevalent in Fontan patients (9, 23, 24, 108). Hearing loss may result from hypo-perfusion, peri-operative injury, or bolus furosemide administration. Screening may aid in early detection of hearing deficiencies.

We found a prevalence of sleep disordered breathing of 36% in Fontan patients (113–115). This is on the high end of estimates for the general population (131). Interestingly, in (biventricular) congestive heart failure, the pathophysiology of heart failure and sleep disordered breathing is closely related (132). Sleep disordered breathing may increase pulmonary vascular resistance and impair hemodynamics of the Fontan circulation (113). Conversely, congestive heart failure may cause noctural soft-tissue swelling of the upper airway (similar to the pathophysiology of orthopnea) (132). CPAP is often the first choice of treatment for sleep-disordered breathing. Establishing CPAP thresholds for Fontan patients may be more difficult due to the hemodynamics of the Fontan circulation, as high airway pressure may increase pulmonary vascular resistance (114, 115). Sleep-disordered breathing is a diagnosis that needs to be considered when a Fontan patient presents with symptoms of sleep-disordered breathing, e.g., excessive daytime sleepiness, snoring, and morning headaches. Safe thresholds for CPAP can be established by monitoring using echocardiography or cardiac catheterization (114, 115).

Due to the systematic nature of our review, we also found various minor abnormalities in multiple organ systems. These abnormalities, such as dental abnormalities, abnormalities of the autonomic nervous system, etc. may be of limited clinical consequence, or data on these problems is currently of insufficient quality of evidence to affect clinical routine.

### Pathophysiology of Organ Abnormalities

#### Cyanosis in Early Infancy

Cyanosis occurs in early infancy and may persist following Fontan palliation (due to residual right-to-left shunting). Cyanosis stimulates erythrocyte production (which in excess may impair capillary perfusion), induces metabolic stress, and activates hypoxia-mediated biological pathways, which may lead to organ dysfunction (122, 133). Importantly, cyanosis has been implicated in the pathogenesis of neuro-endocrine neoplasms in Fontan patients (91–96). Other Fontan sequelae in which cyanosis has been implicated include structural brain abnormalities (10, 11), renal dysfunction (53, 55), and delayed puberty (83).

#### Fontan Physiology

Fontan physiology is characterized by decreased cardiac output and increased central venous pressure. Decreased cardiac output may lead to decreased perfusion of organs. This has been implicated as a cause of among others renal dysfunction (41, 48), decreased BMD (103), and decreased brain volumes (11, 19).

Increased central venous pressure may lead to venous congestion and hamper lymphatic drainage. Impaired lymphatic drainage is considered an important factor in the pathogenesis of plastic bronchitis and PLE (7). Impaired lymphatic drainage may occur in any organ system and may play a role in the lymphopenia seen in Fontan patients (78). Venous congestion may play a role in, among others, the development of pituitary congestion and renal dysfunction (18, 54).

#### Thrombo-Embolic Events

Following Fontan completion, patients remain at increased risk for thrombo-embolism (8). Stroke is considered an important cause of CNS abnormalities, and may relate to other abnormalities described in this review. Micro-emboli may impair end-organ function of among others kidney and spleen (134, 135). The risk of thrombo-embolic events should be minimized during follow-up. Anti-coagulation therapy is commonly recommended, although current guidelines acknowledge the evidence for benefit is limited, and indicate anti-coagulation only for patients with a history of thrombo-embolism or arrhythmia (124, 125).

#### Iatrogenic Factors

Several iatrogenic factors may relate to the abnormalities described in this systematic review. Fontan patients typically undergo multiple cardiothoracic procedures in childhood. Peri-operative conditioning, which may include cardiopulmonary bypass, exposes the body to significant oxidative stress (136, 137). This peri-operative injury has been implicated in the pathogenesis of structural brain abnormalities (10), renal dysfunction (48), and delayed puberty (83).

Medication use is common in Fontan patients compared to healthy peers (138). Side effects of medication may account for some abnormalities described in this review. For example, bolus administration of furosemide may account for the high prevalence of hearing disabilities (108).

#### Lifestyle

Fontan patients may lead different lifestyles compared to healthy peers (139). They may participate less frequently in physical activities (139). This may contribute to the myopenia and decreased bone mineral density seen in Fontan patients (100). Vitamin D deficiency is common in Fontan patients, which may be influenced by dietary and exercise habits (78, 79). Vitamin D deficiency may contribute to myopenia and decreased BMD in Fontan patients (78, 100). Parathyroid function—which may be abnormal in Fontan patients—is also closely related to vitamin D status. Vitamin D status can safely and effectively be improved for Fontan patients with dietary supplementation (79). However, the effect of dietary supplementation on bone mineral density has not been evaluated.

### Limitations

Despite our study's strengths, some limitations should be considered. No formal quality assessment for articles was performed. Because of our broad and descriptive research question, we anticipated a large variety of study designs. Formal quality assessment tools are usually limited to a specific study design. We took a narrative approach to describing the results of the search, rather than performing meta-analyses, as the differing study designs complicates direct comparisons.

A broad systematic search strategy was necessary to provide a complete overview for our research question. Defining objective exclusion criteria, while providing a clinically relevant overview, proved difficult. Some articles were excluded because they did not relate to the research question. These articles were excluded by author consensus and are provided in the online supplement. Despite these limitations, we provide an extensive overview of abnormalities in multiple organ systems.

## Conclusions

We performed a systematic review of abnormalities in multiple organ systems for patients with a longstanding Fontan circulation. We found abnormalities in multiple organ systems including the brain, kidney, and musculoskeletal system. An overview is provided in Supplementary Table 1. Organ abnormalities may be related to Fontan physiology, cyanosis, iatrogenic factors—such as peri-operative injury or medication use-, or lifestyle-related factors. Based on our results we recommend—in addition to current guidelines-: 1) assessment of renal function should be based on cystatin C in addition to creatinine; 2) health care providers should be vigilant for hypothyroidism, visual or hearing deficits, and sleep disordered breathing in Fontan patients; 3) physical exercise may be employed to improve Fontan cardiovascular function, Fontan-associated myopenia, and bone mineral deficits, among other health benefits. Our findings may aid health care providers and provides suggestions for future research.

## Data Availability Statement

The original contributions presented in the study are included in the article/Supplementary Material, further inquiries can be directed to the corresponding author.

## Author Contributions

ER and VV: methodology, investigation, and writing—original draft. JV: conceptualization, writing—original draft, writing—review and editing, visualization, and supervision. GT: investigation and writing—original draft. CC and FU: writing—review and editing. WH: writing—review and editing and supervision. All authors contributed to the article and approved the submitted version.

## Funding

JV was supported by a research grant from the Dutch Heart Foundation (Grant No. 2013T091).

## Conflict of Interest

The authors declare that the research was conducted in the absence of any commercial or financial relationships that could be construed as a potential conflict of interest.

## Publisher's Note

All claims expressed in this article are solely those of the authors and do not necessarily represent those of their affiliated organizations, or those of the publisher, the editors and the reviewers. Any product that may be evaluated in this article, or claim that may be made by its manufacturer, is not guaranteed or endorsed by the publisher.
