# Supplementary material for: Fontan Circulation Associated Organ Abnormalities Beyond the Heart, Lungs, Liver, and Gut: A Systematic Review
Source: Front Cardiovasc Med. 2022 Mar 22;9:826096. doi: 10.3389/fcvm.2022.826096 (PMC8981209; doi:10.3389/fcvm.2022.826096)
Supplement: Supplementary file 1 [file Table_1.pdf]

Table S1. Study results. Articles are sorted by organ system and study design. Data is presented as mean ± standard deviation, median [interquartile range], or count (percentage) unless stated otherwise. Ages and follow-up durations are shown in years unless stated otherwise.

Ref Nr.	Study design	N (Fontan patients)	Age	Time since Fontan	Age at Fontan	Follow-up	Control population	Main findings
Neurology								
Central nervous system imaging abnormalities								
**(9)**	Cross-sectional study	27	5 to 7.5	-	2.3 range 1.7-5.3	-	-	- The study found ischemic changes in watershed areas in 7/20 (35%) patients<br>- Full-scale IQ was lower in patients with abnormalities (97 vs 69, p = .045)
**(10)**	Cross-sectional study	156	14.5 ± 2.9	-	-	-	111 Healthy controls	- Abnormalities on neuro-imaging were more prevalent for Fontan patients versus controls (66% versus 6%)<br>- 13% of patients had evidence of previous stroke, of which 40% were previously undiagnosed<br>- Imaging abnormalities related to neuropsychological test scores reported by patients' parents, but not self-reported scores
**(11)**	Cross-sectional study	128	14.6 ± 2.9	-	-	-	48 Healthy controls	- Grey matter volume and cortical thickness is reduced in Fontan patients
**(12)**	Cross-sectional study	20	15.8 ± 1.1	-	-	-	36 Healthy controls	- Fontan patients have widespread higher T2-relaxation values (a sign of tissue injury) on neuro-imaging
**(13)**	Cross-sectional study	102	15 ± 3	-	-	-	47 Healthy controls	- Evidence of widespread altered white matter microstructure on diffusion tensor imaging<br>- Abnormalities in several anatomical tracts related to full-scale IQ and processing speed
**(14)**	Cross-sectional study	27	16 ± 1	-	-	-	31 Healthy controls	- The study found decreased gray matter density in several regions<br>- Prefrontal, occipital and temporal gray matter density relates to mood and cognitive ability
**(15)**	Cross-sectional study	27	15.7 ± 1.2	-	-	-	35 Healthy controls	- Multiple brain sites in U-CHD showed increased diffusivity values on diffusion tensor imaging<br>- Increased diffusivity may relate to tissue injury, specifically myelin changes.
**(16)**	Cross-sectional study	100	23 ± 8	-	4.9 [3.8 - 6.2]	-	41 Healthy controls	- Structural brain injury in 100% of subjects<br>- Only white matter injury was associated with worse paired associate learning.
**(17)**	Cross-sectional study	17	15.5 ± 6.1	-	-	-	19 Healthy controls	- Fontan patients have decreased grey matter volume
**(18)**	Cross-sectional study	25	16 [15.0 - 17.0]	-	-	-	38 Healthy controls	- Total brain volume is reduced for Fontan patients (1.1 ± 0.1 L versus 1.2 ± .1 L)<br>- Brain volumes are associated with cognitive and memory deficits

Ref Nr.	Study design	N (Fontan patients)	Age	Time since Fontan	Age at Fontan	Follow-up	Control population	Main findings
**(19)**	Cross-sectional study	23	15.9 ± 1.3	-	-	-	37 Healthy controls	- Fontan patients have reduced volumes of the caudate nuclei<br>- Caudate volumes are associated with mild depressive symptoms and cognitive impairment
**(20)**	Prospective cohort study	18	9 ± 2	-	2.2 ± 0.3	8	9 CHD patients	- Fontan patients have reduced total brain volume compared to patients with transposition of the great arteries<br>- Full-scale IQ correlated with total brain volume (9 patients with transposition of the great arteries included)
**(21)**	Cross-sectional study	25	15.9 ± 1.2	-	-	-	38 Healthy controls	- Fontan patients have reduced right, but not left, hippocampal volume<br>- Hippocampal volumes correlated with working memory scores
**(22)**	Cross-sectional study	40	9 [8 - 12]	-	-	-	40 Healthy controls	- Fontan patients have increased pituitary volumes<br>- Pituitary volumes related to central venous pressure
**(23)**	Cross-sectional study	15	5 to 12	-	-	-	-	- 42% of patients had moderately to severely impaired neurological function, among which gross and fine motor dysfunction and seizures
**(24)**	Cross-sectional study	34	4.6 ± 0.3	-	-	-	85 Children with BiV CHD	- Motor function is impaired for Fontan patients, compared to BiV CHD and reference values
Cerebral hemodynamics								
**(25)**	Cross-sectional study	19	6.7 ± 2.6	-	-	-	32 Healthy controls	- Fontan patients have an increased cerebral-to-systemic cardiac output ratio compared to patients with structurally normal hearts<br>- This might reflect a compensatory mechanism to preserve cerebral blood flow in a context of reduced cardiac output
**(26)**	Cross-sectional study	18	11.8 + 2.8	8.7 ± 3.3	3.1 ± 0.7	-	20 Healthy controls	- Cerebral local tissue oxygenation, assessed by near infrared spectroscopy, decreases during exercise for Fontan patients<br>- This response is not seen for healthy controls
**(27)**	Cross-sectional study	10	28 ± 2	-	7 ± 8	-	12 Healthy controls	- Fontan patients have an impaired response in cerebral blood flow to cognitive stimuli, compared to controls
The autonomic nervous system								
**(28)**	Prospective cohort study	50	22.7 ± 3.6	12.6 ± 3.9	-	4	10 Healthy controls	- Fontan patients have increased sympathetic tone compared to controls (circulating norepinephrine 380 ± 30 versus 171 ± 26 pg/mL)<br>- A model of peak oxygen uptake and norepinephrine levels predicted event-free survival
**(33)**	Prospective cohort study	63	13.2 ± 4.7	6.7 ± 5.1	-	1.8	44 controls	- Fontan patients exhibit severely impaired autonomic sympathetic tone, assessed by heart rate variability, circulating levels of norepinephrine, pharmacological studies, and arterial baroreflex response.<br>- Sympathetic tone was not related to clinical characteristics<br>- No beneficial effect of angiotensin converting enzyme (ACE) inhibitor therapy on sympathetic tone was observed during follow-up.

Ref Nr.	Study design	N (Fontan patients)	Age	Time since Fontan	Age at Fontan	Follow-up	Control population	Main findings
**(29)**	Cross-sectional study	67	23 ± 7	11 ± 6	-	-	27 Healthy controls	- Fontan patients have higher sympathetic tone (circulating norepinephrine 423 ± 201 versus 283 ± 97, p < .05)
**(30)**	Cross-sectional study	18	25 ± 1	-	6 ± 1	-	23 Healthy controls	- Fontan patients have elevated muscle sympathetic nerve activity (40 ± 5 vs 27 ± 3 bursts per 100 heartbeats) and normal sympathetic baroreflex function
**(31)**	Cross-sectional study	8	28.6 ± 9.7	18.1 ± 4.8	-	-	12 Healthy controls	- Fontan patient have reduced heart rate variability, a measure of cardiac autonomic nervous activity, but normal heart rate recovery following exercise<br>- Cardiac autonomic nervous activity may be normal in Fontan patients. Heart rate variability may be explained by reduced vascular capacitance
**(32)**	Cross-sectional study	29	28.5 ± 7.3	-	-	-	57 Healthy controls	- Fontan patients have reduced heart rate variability, a measure of autonomic activity
Central nervous system infections								
**(34)**	Case report	1	25	24	1	-	-	- Fontan patients may be at risk of cerebral abscesses due to shunting
**(35)**	Case report	1	28	-	-	-	-	- Fontan patients may be at risk of cerebral abscesses due to shunting
**(36)**	Case report	1	15	-	3	-	-	- Fontan patients may be at risk of cerebral abscesses due to shunting and cerebral focal areas of tissue hypoxia
**(37)**	Case report	1	11	7	4	-	-	- Fontan patients may be at risk of cerebral abscesses due to shunting<br>- Fontan patients living at high altitude may have increased risk of shunting<br>- Good dental hygiene may prevent bacteremia and serious complications
**(38)**	Cross-sectional study	746	-	-	-	-	-	- Fontan patients have an increased risk of central nervous system infections: HR 3.0 (95% CI 1.1 - 8.2)
Headaches								
**(39)**	Cross-sectional study	54	26 ± 9	-	-	-	-	- 50% of Fontan patients report frequent headaches
**(40)**	Case report	1	19	18	1.3	-	-	- Fontan patients may be at risk for pseudotumor cerebri (elevated intracranial pressure in the absence of intracranial mass lesions)<br>- Fontan patients with refractory headaches should be evaluated for increased intracranial pressure
Renal system								
**(41)**	Retrospective cohort study	328	26 ± 6.5	-	-	7 ± 5	-	- 20% of adult Fontan patients have renal dysfunction based on creatinine-based GFR estimates<br>- 1% of patients had severe renal dysfunction<br>- 10-Year survival and 10-year freedom from death and transplantation did not differ between patients with and without renal dysfunction.<br>- Mean GFR did not change for patients with renal dysfunction over a 8 ± 5.5 year follow-up

Ref Nr.	Study design	N (Fontan patients)	Age	Time since Fontan	Age at Fontan	Follow-up	Control population	Main findings
**(42)**	Cross-sectional study	81	28.4 ± 9.3	-	-	-	-	- Using invasive renal function measurements, 53% of patients have kidney dysfunction. 2% had severe kidney dysfunction<br>- Invasive renal function measurements correlated modestly with creatinine-based GFR estimations<br>- Creatinine-based kidney function estimates might not be accurate in the Fontan population due to decreased muscle mass<br>- Renal dysfunction may be an early sign of Fontan failure and a marker of end organ damage.
**(43)**	Prospective cohort study	70	30.7 ± 9.8	-	-	707 d [371 - 942]	20 Age- and sex matched controls	- Cystatin C based GFR estimates were significantly lower in Fontan patients compared to controls.<br>- 12.9% of Fontan patients had renal dysfunction based on Cystatin C estimates compared to none of the controls<br>- 22.9% of Fontan patients had renal dysfunction based on creatinine estimates compared to 15% of the controls<br>- Fontan patients have a higher prevalence of microalbuminuria (33.9%) compared to controls (0%), which is indicative of glomerular injury<br>- Cystatin C based GFR estimates, but not creatinine-based ones, were associated with non-elective cardiovascular hospitalization or death
**(44)**	Retrospective cohort study	68	13 [9.0 - 17.3]	11.1 [6.5 - 15.7]	-	-	70 healthy children similar in age and sex	- Using different creatinine and cystatin C-based formulae, GFR estimates were comparable between Fontan patients and healthy controls<br>- 10 % of Fontan subjects had renal dysfunction using a combined creatinine and cystatin C formula<br>- 10% of the Fontan patients presented with proteinuria compared to 4.7% in controls (p = .27)
**(45)**	Case control study	5	-	-	-	-	48 non-hypoxemic CHD patients	- Patients with cyanotic CHD had higher rates of proteinuria compared to those with acyanotic CHD.<br>- 24-hour proteinuria did not differ between Fontan patients and those with other cyanotic CHD diagnoses
**(46)**	Prospective cohort study	283	16 ± 8	-	5 ± 6	3.7 ± 2.1	-	- Renal dysfunction was predictive of unscheduled hospitalizations in the adult Fontan population, but not in children
**(47)**	Retrospective cohort study	50	Range 20 to 53	-	-	-	18 Non-Fontan single ventricle patients	- Renal function was more favorable in young adults, compared to older adults
**(48)**	Cross-sectional study	21	15.2 ± 8.8	11.4 ± 6.5	-	-	-	- Microalbuminuria, a marker of renal injury, was found in 43% of patients<br>- Microalbuminuria may be related to increased CVP<br>- Patients using ace-inhibitors had less microalbuminuria compared to those who did not
**(49)**	Retrospective cohort study	67	-	-	-	-	-	- 15 (22%) Fontan patients suffered from renal dysfunction, of which 1 moderate and 1 severe dysfunction.<br>- Patients with renal dysfunction had higher CVP compared to patients without.<br>- African American Fontan patients may be at increased risk for renal dysfunction
**(50)**	Retrospective cohort study	49	49.2 ± 6.4	-	-	4.9 [1.8 - 8.5]	-	- Renal disease is one of the most common extracardiac comorbidities in Fontan patients, with a prevalence of 30.6% in this cohort<br>- The presence of renal disease is an independent predictor of all-cause mortality

Ref Nr.	Study design	N (Fontan patients)	Age	Time since Fontan	Age at Fontan	Follow-up	Control population	Main findings
**(51)**	Cross-sectional study	152	19.8 ± 9.3	14.1 ± 7.6	-	-	-	- 57% of patients had either renal dysfunction (assessed by invasive methods) or proteinuria<br>- Renal was mild in 35% of patients, and moderate in 2%<br>- Microalbuminuria was found in 37% of Fontan patients.
**(52)**	Prospective cohort study	86	-	-	-	270 ± 288 d	-	- Albuminuria was more prevalent among patients with a Fontan circulation, compared to other CHD patients. Other risk factors include cyanosis, systemic right ventricle and lower functional status.<br>- Albuminuria was not associated with an increased risk for adverse outcomes in patients with a Fontan circulation
**(53)**	Retrospective cohort study	98	25.5 ± 8.9	17.2 (median)	9.1 ± 9.0	-	-	- Systolic ventricular dysfunction and decreased oxygen saturation were associated with renal and hepatic dysfunction
**(54)**	Case control study	39	14.8 ± 7.9	-	-	-	29 healthy controls (age 12.7 ± 2.7)	- Microalbuminuria was present in 33% of Fontan patients<br>- Inferior caval vein diameter, as a surrogate for elevated CVP, was significantly larger in Fontan patients compared to controls<br>- Patients with microalbuminuria had higher inferior caval vein diameter compared to those without
**(55)**	Case report	1	14	-	1.6	-	-	- Renal histological abnormalities may be subtle, despite an extensive clinical history of kidney injury. Renal dysfunction may be reversible in these cases.
**(56)**	Case report	1	41	-	27	-	-	- Focal segmental glomerulosclerosis may underlie proteinuria and renal dysfunction in Fontan patients
**(57)**	Case series	2	29 ± 4	-	7.5 ± 5.5	-	-	- Focal segmental glomerulosclerosis could be associated with the Fontan circulation, relating to increased central venous pressure and hypoxia
**(58)**	Retrospective cohort study	426	-	-	2.3 [1.8 – 4.3]	6.6 ± 5.4	-	- Serum creatinine and urea were within normal limits 10 and 15 years postoperatively<br>- Serum creatinine increased from 10 to 15 years post Fontan (0.6 ± 0.2 to 0.8 ± 0.2 mg/dl, p <.001)
**(59)**	Prospective cohort study	280	19 ± 9	-	-	32 ± 22	36 Healthy controls	- Fontan patients had a significantly higher renal resistive index (RRI) than the healthy controls<br>- High CVP, low arterial pressure, higher pulse pressure and low arterial oxygen saturation were independently related to a high RRI.<br>- Renal resistive index was inversely correlated with 24 hour creatinine clearance.<br>- Renal resistive index ≥ 0.81 independently predicts mortality. 5. RRI could be a useful marker of prognosis in Fontan patients.
Muscular system								
**(65)**	Retrospective cohort study	47	< 22	-	3.9 ± 1.9	-	165 Healthy controls	- Fontan patients had lower lean body mass, skeletal muscle mass, and higher percent body fat.
**(66)**	Cross-sectional study	13	29.0 ± 5.9	-	-	-	12 age-matched healthy controls	- Skeletal muscle mass were significantly decreased in Fontan patients compared to that in controls<br>- Serum ghrelin levels (an orexigenic hormone) were also decreased in Fontan patients, which may affect body composition

Ref Nr.	Study design	N (Fontan patients)	Age	Time since Fontan	Age at Fontan	Follow-up	Control population	Main findings
**(26)**	Cross-sectional study	18	11.8 ± 2.8	8.7 ± 3.3	3.1 ± 0.7	-	18 Healthy controls	- Cerebral local tissue oxygenation, assessed by near infrared spectroscopy, decreased during exercise for Fontan patients<br>- This response is not seen for healthy controls<br>- Cerebral deoxygenation during exercise may play a role in the impaired exercise performance of Fontan patients.
**(61)**	Cross-sectional study	16	30 ± 2	16 ± 3	10 ± 2	-	8 age- and sex-matched controls	- Skeletal muscle mass was significantly reduced compared to controls<br>- Skeletal muscle mass Z-score for Fontan patients was −1.5 ± 0.2<br>- 25% of patients had muscle wasting in the sarcopenic range<br>- Muscle aerobic capacity, measured by the rate of post-exercise phosphocreatine resynthesis, was significantly impaired in Fontan patients versus controls
**(60)**	-	-	-	-	-	-	-	This is an erratum for (61). An author was erroneously unreported
**(62)**	Cross-sectional study	50	11.5 range 5.1-33.5	-	-	-	Z-scores based on 992 healthy participants	- Lean mass Z-scores were lower in Fontan patients compared to controls.<br>- Lean mass was not associated with age or Fontan characteristics<br>- Leg lean mass Z-scores were lower in vitamin D deficient versus sufficient Fontan participants
**(63)**	Cross-sectional study	28	26 ± 7	-	-	-	-	- Skeletal muscle mass for Fontan patients was reduced compared with reference data<br>- Patients with reduced skeletal muscle had lower oxygen pulse (a measure of exercise capacity)
**(64)**	Cross-sectional study	40	25.5 ± 7.0	-	4.4 ± 3.8	-	-	- Muscle mass may be estimated from MRI images obtained for the evaluation of liver in Fontan patients<br>- Parameters of muscle mass was significantly lower in the male Fontan population compared with the healthy historic cohort, but was similar for women<br>- Skeletal muscle index at L3 level was positively correlated with exercise capacity
**(67)**	Cross-sectional study	43	12.2 ± 3.9	-	-	-	43 age- and sex-matched controls	- Adult Fontan patients had impaired isometric knee extension strength in comparison with the controls. This was not seen for adolescent patients.<br>- Leg lean mass was highly correlated to isometric knee extension strength
**(68)**	Cross-sectional study	43	12.8 range 5.1-33.5	14.4 ± 12.3	-	-	Z-scores based on >700 reference participants	- Calf muscle area Z-scores were lower in the Fontan participants and lower calf muscle area Z-scores were associated with bone structures
**(70)**	Cross-sectional study	11	-	-	-	-	10 Healthy controls	- Fontan patients have decreased cardiopulmonary exercise capacity, decreased lung function, and decreased muscle strength, compared to healthy peers<br>- Respiratory and skeletal muscle weakness did not differ between Fontan patients and other CHD patients
**(71)**	Cross-sectional study	64	9.0 ± 1.7	5.7 ± 2.0	3.3 ± 1.4	-	-	- Fontan patients had similar strength scores to their peers but perform less moderate-to-vigorous activity per day compared to controls (12 ± 17 min/day less, $p < .001$).<br>- Physical endurance and flexibility for Fontan patients were lower than peers.
**(74)**	Cross-sectional study	50	21 ± 7	9.6 ± 4.3	11.1 ± 6.5	-	15 Healthy controls	- Fontan patients have reduced skeletal muscle blood supply, which may attenuate oxygen re-saturation after exercise<br>- These factors may relate to diminished exercise capacity in Fontan patients

Ref Nr.	Study design	N (Fontan patients)	Age	Time since Fontan	Age at Fontan	Follow-up	Control population	Main findings
**(75)**	Cross-sectional study	30	18 [15-24]	10 [8-15]	8 ± 3	-	27 Healthy controls	- Fontan patients have a reduced cross-sectional area of the thigh, lower walked distance and lower handgrip strength<br>- Muscle sympathetic nerve activity and norepinephrine concentrations (markers of sympathetic tone) were higher in Fontan patients<br>- Fontan patients have blunted forearm blood flow and forearm vascular conductance compared to controls
**(76)**	Cross-sectional study	43	12.2 ± 3.9	-	-	-	43 age- and sex-matched controls	- Fontan patients had a blunted increase in muscle oxygen extraction during exercise compared to controls.<br>- Following exercise, patients exhibited a slower tissue oxygenation re-saturation than controls<br>- Differences in oxygenation responses to exercise were seen between adolescent patients and healthy peers, but not between pediatric patients and healthy peers
**(69)**	Case control study	17	-	-	-	-	124 Healthy controls	- Fontan patients had lower hand grip strength compared to healthy controls and other CHD diagnoses
**(72)**	Case control study	13	17.7 range 12.7-26	-	-	-	Sex-specific Z-scores	- Leg lean muscle mass relates to cardiac index and increases in cardiac index during exercise
**(73)**	Other	11	32 ± 2	21 ± 1	11 ± 4	17m	-	- Resistance training increased muscle strength and peak oxygen uptake, following 20 weeks of training.
Endocrinology								
**(22)**	Cross-sectional study	40	9.3 (8.3 - 11.5)	-	3.3 [1.6 - 5.7]	-	40 healthy controls	- The volume of the pituitary is larger in Fontan patient, in contrast to most brain structures<br>- This may relate to pituitary edema
Thyroid-axis								
**(77)**	Retrospective cohort study	37	8.0 ± 3.9	5.2 ± 4.0	-	-	-	- 33% of the patients had subclinical hypothyroidism<br>- Low FT3 levels were associated with decreased systolic and diastolic function
**(50)**	Retrospective cohort study	49	49.2 ± 6.4	-	-	4.9 [1.8 - 8.5]	-	- 55.1% of Fontan patients were diagnosed with hypothyroidism
**(39)**	Cross-sectional study	54	26 ± 9	-	-	-	-	- 13% of Fontan patients were diagnosed with hypothyroidism
Parathyroid axis								
**(44)**	Retrospective cohort study	68	13 [9.0 - 17.3]	11.1 [6.5 - 15.7]	25m [21 - 39]	-	70 healthy controls	- Parathyroid hormone levels were highly increased in Fontan patients compared to healthy controls
**(78)**	Cross-sectional study	265	12.8 [9.5 - 16.4]	-	2.6 [2.0 - 3.4]	-	-	- Parathyroid hormone levels do not relate to exercise capacity<br>- The prevalence of vitamin D deficiency in Fontan patients is 81%
**(79)**	Retrospective cohort study	27	8.1 ± 5.3	-	2.6 ± 1.1	5.8 m ± 3.7	-	- The prevalence of vitamin D deficiency in Fontan patients is 70%<br>- Vitamin D supplementation can increase vitamin D levels and decrease parathyroid levels

Ref Nr.	Study design	N (Fontan patients)	Age	Time since Fontan	Age at Fontan	Follow-up	Control population	Main findings
Renin-angiotensin-aldosterone system								
**(81)**	Prospective cohort study	44	29.2 ± 10.7	23.8 ± 5.1	7.5 ± 6.3	1	32 matched controls	- Fontan patients have increased serum renin, angiotensin II and aldosterone levels compared to healthy controls
**(59)**	Prospective cohort study	280	19 ± 9	-	4.9 ± 5.8	32m ± 22	36 healthy controls	- There is a correlation between plasma renin activity and renal vascular resistance
**(140)**	Retrospective cohort study	169	17 ± 8	-	-	2.1	-	- 30% of Fontan patients have hyponatremia<br>- Plasma natrium is an independent predictor for unscheduled rehospitalization
**(80)**	Cross-sectional study	12	11 [4 - 22]	2 [0.5 - 6]	-	-	33 healthy controls	- Fontan patients have increased serum renin, angiotensin II and aldosterone levels compared to healthy controls
Sex hormones								
**(83)**	Cross-sectional study	299	13.9 [11.3 - 16.1]	-	-	-	-	- 58% of the Fontan patients had a delay in one of the Tanner stage parameters<br>- There was a median delay of 1.5-2 years between Fontan patients and the normal population in achieving the Tanner stages<br>- Multiple surgeries with cardiopulmonary bypass in early childhood may result in hypothalamic-pituitary gonadal axis malfunction
Appetite-related hormones								
**(66)**	Cross-sectional study	13	29.0 ± 5.9	-	-	-	12 healthy controls	- In the Fontan patients plasma ghrelin levels were lower than those in controls<br>- The authors proposed that lower ghrelin levels might relate to the risk of heart failure<br>- Ghrelin has a favorable effect on cardiovascular function and is seen as potentially therapeutic target
Metabolism								
**(29)**	Prospective cohort study	67	23 ± 7	-	-	11 ± 6	27 Healthy volunteers	- Fontan patients have decreased plasma glucose; increased insulin, C-peptide, and HbA1c<br>- Fontan patients have abnormal glucose tolerance<br>- Fontan patients have lower total, LDL, and HDL cholesterol
**(46)**	Prospective cohort study	283	16 ± 8	-	5 ± 6	9 ± 8	-	- Abnormal glucose metabolism predicts mortality and unplanned hospitalization<br>- Cholesterol and glucose metabolism did not differ between adult and pediatric patients<br>- Total cholesterol does not predict outcomes
**(59)**	Prospective cohort study	280	19 ± 9	-	5 ± 6	32 ± 22 m	36 healthy volunteers	- Abnormal glucose metabolism relates to renal vascular resistance
**(85)**	Prospective cohort study	175	20 ± 7	-	-	7.1 ± 3.9	28 healthy volunteers	- 34% of Fontan patients have impaired glucose tolerance<br>- 5% of Fontan patients have diabetes mellitus<br>- Oral glucose tolerance and HbA1c decreases over a follow-up of 6.5 ± 2.7 years
**(81)**	Prospective cohort study	44	29.2 ± 10.7	-	7.5 ± 6.3	1	-	Adiponectin, a hormone of lipid catabolism, was increased in Fontan patients compared to controls

Ref Nr.	Study design	N (Fontan patients)	Age	Time since Fontan	Age at Fontan	Follow-up	Control population	Main findings
**(141)**	Prospective cohort study	20	23.1 ± 5.1	18.8 ± 5.2	-	-	20 age- and sex-matched biventricular controls	- Serum concentration of several amino acids, among which glutamic acid and hydroxyproline, are increased, whereas other amino acids are decreased (among others taurine, asparagine, and threonine)<br>- The methionine sulfoxide to methionine ratio is decreased in Fontan patients compared to healthy controls and negatively correlates with exercise capacity
**(86)**	Retrospective cohort study	88	14.8 [10.6 - 20.0]	-	2.1 [1.8 - 3.2]	-	-	- Total cholesterol, LDL cholesterol, non–HDL cholesterol, and HDL cholesterol levels were significantly lower in Fontan patients than controls<br>- Cholesterol levels were below the 25th percentile for age and sex for total cholesterol in 82% of patients, for LDL-C in 76%, for non–HDL-C in 67%, and for HDL-C in 57%<br>- Hypocholesterolemia may relate to inflammation
**(87)**	Cross-sectional study	20 (65% male)	23.1 ± 5.1	18.8 ± 5.2	-	-	20 healthy controls	- Fontan patients have abnormal levels of phospholipid subtypes: lower levels of phosphatidylcholines, sphingomyelins, and higher levels of acylcarnitines<br>- Specific changes in the phospholipids metabolism may relate to a heart failure phenotype.
**(84)**	Cross-sectional study	21 (62% male)	13.6 [11.4 - 17.3]	11.4 [8.5 - 14.2]	-	-	21 healthy control patients	- Fontan patients have lower total cholesterol<br>- Fontan patients have lower levels of different cholesterol subtypes compared to published reference values
**(88)**	Cross-sectional study	16 (50% male)	9.7 [4.9 - 17.8]	-	33m [15 - 50]	-	10 healthy controls	- Amino acid-related metabolites differ between Fontan patients and controls<br>- Metabolite profiles can distinguish between Fontan patients with and without heart failure
Neoplastic disease								
**(90)**	Prospective cohort study	180	21.4 ± 7.4	-	-	9.1 [5.2 - 14.5]	-	- Cancer was the cause of death in 3% of the Fontan population. Comparisons to controls are complicated by differing mortality rates.
**(91)**	Retrospective cohort study	7	Range 13-38	21.4 range 10.4 - 29.7	-	-	-	- The prevalence of pheochromocytoma and paraganglioma is 2.5% among Fontan patients
**(92)**	Case report	1	23	11	12	-	-	- Case report of a pheochromocytoma in a 23-year-old female Fontan patient
**(93)**	Case report	1	22	-	2 m	-	-	- Case report of a pheochromocytoma in a 14-year-old female Fontan patient
**(94)**	Case report	1	11	-	4	-	-	- Case report of a paraganglioma in a 11-year-old female Fontan patient
**(95)**	Case report	1	18	-	1	-	-	- Case report of a paraganglioma in an 18-year-old male Fontan patient
**(96)**	Case series	2	18 ± 5	-	1	-	-	- This case series detailed two male Fontan patients with a paraganglioma
**(98)**	Case series	2	14 ± 4	-	4.5 ± 1.5	-	-	- This case series detailed two cases of gastrointestinal neuroendocrine tumors (NET) in Fontan patients

Ref Nr.	Study design	N (Fontan patients)	Age	Time since Fontan	Age at Fontan	Follow-up	Control population	Main findings
**(97)**	Other	8	27.8 ± 12.6	-	-	-	-	- This study found strong link between cyanotic congenital heart disease and an increased risk of pheochromocytoma and paraganglioma
Bone								
**(101)**	Retrospective cohort study	194	3.4 [1.6 - 8.2]	-	1.3 [1.1 - 1.9]	-	-	- Prevalence of scoliosis in Fontan patients is higher compared to the general population (9.8% versus 2-3%)<br>- The risk of scoliosis increases with age, independently of previous thoracotomies<br>- Females seemed to be at higher risk for developing scoliosis, with a female to male ratio of 5:3
**(104)**	Cross-sectional study	29	-	-	-	-	-	- Bone mass was decreased in Fontan patients, compared to both healthy references and other CHD diagnoses
**(99)**	Cross-sectional study	28	26 ± 7	-	-	-	-	- 29% of Fontan patients had bone mineral density in the osteopenic range and 4% were in osteoporotic range<br>- Parathyroid hormone levels were increased compared to references (6.1 ± 3.5 vs 4 pmol/L, p = .01), probably relating to vitamin D insufficiency.
**(68)**	Cross-sectional study	43	12.8 range 5.1 - 33.5	9.8 range 2 - 26.7	-	-	Z-scores based on >700 reference participants	- Fontan patients had lower trabecular, but not cortical, bone mineral density than controls<br>- Dimensions of the bone cortex were reduced in Fontan patients<br>- Muscoskeletal deficits were not associated with age, Fontan characteristics, parathyroid hormone, or vitamin D levels
**(100)**	Cross-sectional study	64	Range 5 to 18	11.2	2.5	-	-	- Bone mineral density (BMD) was progressively impaired across increasing age groups.<br>- Total body and spine bone mineral density is linearly related to the patients age<br>- Vitamin D levels also decreased with age but were not correlated with bone mineral density deficits.<br>- Lateral tunnel Fontan patients had a lower total body BMD, compared to extra-cardiac Fontan patients
**(39)**	Cross-sectional study	54	26 ± 9	-	-	-	-	- 22% of patients (n=12) in this cohort were diagnosed with scoliosis.
**(102)**	Cross-sectional study	10	12.1 ± 1.77	>5 years	-	-	11 healthy controls	- Fontan patients have bone mineral density in the lower ranges of the normal range for age and sex<br>- Significant reductions in bone density was seen in the radius, a non-weight bearing bone, compared to the tibia, a weight bearing bone. This may suggest that non-weight bearing bones are more affected in Fontan patients<br>- Vitamin D levels were at the lower end of reference values
**(103)**	Cross-sectional study	15	5.6 range 3.1 - 13.1	1.5 range 0.1 - 12.1	3.6 range 1 - 5.6	-	-	- Bone specific alkaline phosphatase, a biomarker produced by osteoblasts, is reduced in the serum of Fontan patients compared to reference z-scores.
**(78)**	Retrospective cohort study	265	12.8 [9.5 - 16.4]	-	2.6 [2.0 - 3.4]	-	-	- Vitamin D insufficiency was found in 64 (19%) of Fontan patients<br>- Patients with high exercise capacity (peak oxygen uptake > 80% of predicted) were predominantly vitamin D sufficient (92% versus 69%, p <.001)
Immune system								

Ref Nr.	Study design	N (Fontan patients)	Age	Time since Fontan	Age at Fontan	Follow-up	Control population	Main findings
**(81)**	Prospective cohort study	44	29.2 ± 10.7	23.8 ± 5.1	7.5 ± 6.3	1	32 matched controls	- Tumor Necrosis Factor-α, Interleukin-6, Growth Derived Factor-15 and β2-macroglobulin were significantly elevated in Fontan patients compared to healthy controls
**(105)**	Retrospective cohort study	73	27.8 ± 8.4	19.6 ± 5.7	-	3.6 ± 1.8	-	- The prevalence of lymphopenia is 32%<br>- There is no age difference between patients with and without lymphopenia<br>- Lymphopenia in Fontan patients may be related to Fontan associated liver disease
**(106)**	Retrospective cohort study	15	10.2 [3.8 - 26.9]	6.5 [0.7 - 22.1]	-	-	-	- The prevalence of lymphopenia is 26.7%<br>- Lymphopenia in Fontan patients may be related to Fontan associated liver disease
**(107)**	Retrospective cohort study	178	10.8 [7 - 16]	7.8 [4 - 13.7]	-	-	-	- The prevalence of lymphopenia is 12%<br>- The proportion of patients with lymphopenia is significantly higher in patients who underwent Fontan completion over 10 years ago<br>- Lymphopenia in Fontan patients may be related to (subclinical) enteric lymph loss
**(78)**	Retrospective cohort study	265	12.8 [9.5 - 16.4]	-	2.6 [2.0 - 3.4]	-	-	- Lymphopenia in Fontan patients may be related to overall cardiovascular function
Auditory system								
**(108)**	Retrospective cohort study	75	-	-	-	-	-	- 11 Fontan patients (17.2%) were reported to have reduced hearing during follow-up
**(23)**	Cross-sectional study	15	range 5 - 12	-	-	-	-	- Two of the studied patients (13%) were reported to have reduced hearing
**(24)**	Cross-sectional study	34	4.6 ± 0.3	-	-	-	85 children with BiV CHD	- None of the studied patients (0%) were reported to have reduced hearing
**(9)**	Cross-sectional study	27	range 5 - 7.5	-	2.3 range 1.7 - 5.3	-	-	- One patient (4%) had a sensorineural hearing deficit and required a hearing aid
Reproductive system								
**(109)**	Retrospective cohort study	11	Range 19 - 24	-	-	-	-	- 5 out of 8 placentas had low weight for gestational age (SGA)<br>- All placentas had some form of hypoxic lesions, which may be related to the Fontan circulation<br>- Chronic hypoxemia, high CVP and low cardiac output may play a part in the development of poor placental health and therefore in pregnancy outcomes in Fontan patients
**(111)**	Cross-sectional study	72	19.5, range 12 to 34	15.2	4.3	-	-	- Most (69%) female Fontan patients have normal menstrual patterns<br>- Menstrual abnormalities included oligo-menorrhea, amenorrhea, and menorrhagia
**(112)**	Cross-sectional study	54	28 ± 3	-	5 ± 3	-	-	- The prevalence of erectile dysfunction did not differ between patients and controls<br>- Overall sexual satisfaction was lower in Fontan patients (8.3 ± 1.9 versus 9.5 ± 0.8 out of 10, p <.001)

Ref Nr.	Study design	N (Fontan patients)	Age	Time since Fontan	Age at Fontan	Follow-up	Control population	Main findings
**(110)**	Case series	7	27.5 ± 3.2	-	8.6 ± 7.3	-	-	- Placental weight is reduced in pregnant Fontan patients (median weight for gestational age percentile 10 to 15)<br>- Prominent subchorionic fibrin deposition was a consistent feature in all placentas
Sleep-disordered breathing								
**(113)**	Retrospective cohort study	22	29 ± 10.1	-	-	-	-	- 77% (n=17) of Fontan patients that had undergone a polysomnography, were diagnosed with sleep-disordered breathing<br>- Sleep-disordered breathing may be common in Fontan patients, and may be a potentially modifiable risk factor for outcomes
**(115)**	Case series	4	26.5 ± 2.2	-	-	-	-	- Continuous positive airway pressure therapy to treat sleep-disordered breathing in Fontan patients can be established while monitoring hemodynamics during cardiac catheterization.
**(114)**	Case report	1	29	-	-	-	-	- Continuous positive airway pressure therapy to treat sleep-disordered breathing in Fontan patients can be established while monitoring with echocardiography
Dermatology								
**(116)**	Cross-sectional study	51	27.3 [18.3 - 53.3]	-	-	-	10 Healthy controls	- The prevalence of lower limb venous reflux was significantly greater in the Fontan population compared to healthy controls<br>- Clinical signs of chronic venous insufficiency were not more common in Fontan patients than in controls
**(39)**	Cross-sectional study	54	26 ± 9	-	-	-	-	- This study found varicose veins of the lower limbs in 11 Fontan patients
**(117)**	Case report	1	2	-	-	-	-	- This case report describes a case of a Fontan patient with disturbed wound healing after a neurosurgical procedure<br>- Increased CVP may hamper scalp perfusion
Ophthalmology								
**(24)**	Prospective cohort study	34	-	-	-	-	-	- Vision impairment was found in 2.9% (n=1) of the Fontan patients
**(23)**	Cross-sectional study	15	range 5 - 12	-	-	-	-	- Four children (27%) presented with strabismus
Dental abnormalities								
**(118)**	Cross-sectional study	11	range 3 - 16	-	-	-	268 age- and sex matched healthy children and adolescents	- Oral health of children with a congenital or acquired heart disease was the same as that of the healthy controls<br>- Dental development of Fontan patients was delayed compared to controls (1.1 ± 0.8 years below chronological age)
**(37)**	Case report	1	11	7	-	-	-	- A brain abscess was found in a Fontan patient due to a pathogen commonly found in dental plaque<br>- Good oral hygiene may prevent bacteremia
Gastro-intestinal								

Ref Nr.	Study design	N (Fontan patients)	Age	Time since Fontan	Age at Fontan	Follow-up	Control population	Main findings
**(120)**	Cross-sectional study	15	27.6 [21.8 - 34.6]	-	-	-	15 Healthy controls	- At baseline, Fontan patients had higher systemic vascular resistance<br>- Following food ingestion, healthy controls decreased mesenteric vascular resistance, whereas Fontan patients did not<br>- Fontan patients temporarily decrease leg vascular resistance following food intake, a response not seen in healthy controls

BiV = biventricular, CHD = congenital heart disease, HR = hazard ratio, U-CHD = univentricular congenital heart disease , GFR = glomerular filtration rate, L3 = third lumbar vertebra, FT3 = free triiodothyronine, HbA1c =glycated haemoglobin, LDL = low-density lipoproteins, HDL = high-density lipoproteins.
