# Supplementary material for: Fontan Circulation Associated Organ Abnormalities Beyond the Heart, Lungs, Liver, and Gut: A Systematic Review
Source: Front Cardiovasc Med. 2022 Mar 22;9:826096. doi: 10.3389/fcvm.2022.826096 (PMC8981209; doi:10.3389/fcvm.2022.826096)
Supplement: Supplementary file 2 [file Data_Sheet_1.docx]

**Embase.com**

('Fontan procedure'/exp OR 'cavopulmonary connection'/de OR 'heart single ventricle'/de OR 'Norwood procedure'/de OR (fontan OR single-ventric* OR univentric* OR ((cavopulmonar* OR cavo-pulmonar*) NEAR/3 (connection* OR anastomos* OR shunt*)) OR (norwood NEAR/3 (stage-3 OR stage-iii))):ab,ti,kw) AND ('digestive system'/exp OR 'digestive system disease'/exp OR 'throat disease'/exp OR 'gastrointestinal surgery'/exp OR 'endocrine system'/exp OR 'endocrine disease'/exp OR 'exocrine gland'/exp OR 'pancreas disease'/exp OR 'immune system'/exp OR 'immunopathology'/exp OR 'integumentary system'/exp OR 'skin disease'/exp OR 'musculoskeletal system'/de OR 'musculoskeletal disease'/exp OR 'bone'/exp OR 'cartilage'/exp OR 'fascia'/exp OR 'joint'/exp OR 'ligament'/de OR 'muscle'/de OR 'muscle blood vessel'/de OR 'neuromuscular system'/de OR 'smooth muscle'/exp OR 'sphincter'/exp OR 'tendon'/exp OR 'striated muscle'/de OR 'skeletal muscle'/exp OR 'nervous system'/exp OR 'neurologic disease'/exp OR 'nervous system development'/exp OR 'sensory system'/exp OR 'sensory dysfunction'/exp OR 'stomatognathic system'/exp OR 'mouth disease'/exp OR 'urogenital system'/exp OR 'urogenital tract disease'/exp OR 'neoplasm'/exp OR 'metabolism'/exp OR 'metabolic disorder'/exp OR 'cholesterol'/exp OR ‘urogenital system function and reproduction’/exp OR (organ* OR ((digestive* OR alimentar*) NEAR/3 (system* OR tract* OR canal*)) OR gastrointest OR intest* OR gut OR guts OR anus OR anal OR esophag* OR oesophag* OR gastr* OR cricopharyng* OR foregut* OR rectum OR rectal OR stomach* OR colon* OR colorect* OR cecum* OR caecum* OR coecum* OR appendi* OR duoden* OR ileocec* OR ileum* OR jejunum* OR midgut* OR pancrea* OR biliar* OR bile-duct* OR gallbladder* OR adrenal OR (suprarenal* NEAR/3 gland*) OR gonad* OR ovar* OR testis OR testes OR testicular* OR genital* OR gamete* OR muellerian-duct* OR hypophys* OR ((endocrine* OR neuroendocrine*) NEAR/3 (system*)) OR pancrea* OR langerhans* OR paraganglion* OR parathyroid* OR thymus* OR thyroid* OR pituitar* OR adenohypophys* OR neurohypophys* OR exocrine* OR accessory-sex-gland* OR Bartholin* OR breast* OR ((bulbourethral* OR gastrointest* OR mucus* OR paraurethral* OR saliva* OR harder* OR salt* OR sebaceous* OR sweat*) NEAR/3 (gland*)) OR lacrimal* OR tear OR tears OR prostat* OR ((immun* OR reticuloendothelial*) NEAR/3 (system* OR synapse* OR cell*)) OR ((antigen-presenting* OR endothel* OR subendothel* OR mast* OR dendritic* OR inflammator* OR polymorphonuclear* OR foam*) NEAR/3 (cell*)) OR macrophage* OR phagocyt* OR autoimmune* OR rheuma* OR integument* OR tegumkent* OR skin* OR hair* OR nail* OR ((sweat* OR apocrine* OR eccrine* OR sudorifera*) NEAR/3 gland*) OR derma* OR dermis* OR epiderma* OR epidermis* OR scalp* OR wrinkle* OR cutis OR muscle* OR muscul* OR sphincter* OR tendon* OR bone* OR cartilage* OR fascia* OR joint* OR ligament* OR rib OR ribs OR skelet* OR pelvic OR pelvis OR shoulder* OR nerve* OR nervous* OR neuro* OR glia* OR motor-system* OR postganglion* OR preganglion* OR presynap* OR ganglion* OR synapse* OR spinal-cord* OR neural* OR brain* OR forebrain* OR cerebr* OR cerebell* OR meninx OR meninges OR dura OR dural OR epidural OR ((auditory OR gustatory OR vestibular OR visual OR optic*) NEAR/3 system*) OR sensorium* OR neural-analy* OR sense-organ* OR neuroepithel* OR sensorimotor* OR sensory OR ear OR ears OR taste-bud OR olfactory OR eye* OR optic-pathway* OR ocul* OR opthal* OR photoreceptor* OR stomatognathic* OR mouth* OR tooth OR teeth OR dentition* OR dental* OR pharyn* OR gingiva* OR periodont* OR cheek* OR lip OR lips OR palat* OR saliva* OR tongue* OR urogenital* OR genital* OR urinar* OR cloaca* OR genitourinary* OR sex-gland* OR sex-organ* OR gamete* OR gonad* OR placent* OR kidney* OR renal* OR urolog* OR cancer* OR neoplas* OR tumor* OR tumour* OR malign* OR metastas* OR carcino* OR adenocarcino* OR metabol* OR thromb* OR ‘cholesterol’ OR 'renin angiotensin aldosterone system' OR ‘renin’ OR ‘aldosterone’):ab,ti,kw) NOT ((animal/exp OR animal*:de OR nonhuman/de) NOT ('human'/exp)) NOT ([review]/lim OR review:ti) NOT [conference abstract]/lim

**Medline (Ovid)**

(Fontan Procedure/ OR Univentricular Heart/ OR Norwood procedure/ OR (fontan OR single-ventric* OR univentric* OR ((cavopulmonar* OR cavo-pulmonar*) ADJ3 (connection* OR anastomos* OR shunt*)) OR (norwood ADJ3 (stage-3 OR stage-iii))).ab,ti,kf.) AND (exp Digestive System/ OR exp Digestive System Diseases/ OR exp Digestive System Surgical Procedures/ OR exp Endocrine System/ OR exp Endocrine System Diseases/ OR exp Exocrine Glands/ OR exp "Hemic and Immune Systems"/ OR exp Immune System Diseases/ OR exp Integumentary System/ OR exp "Skin and Connective Tissue Diseases"/ OR Musculoskeletal System/ OR Aponeurosis/ OR exp Cartilage/ OR exp Fascia/ OR exp Ligaments/ OR exp Skeleton/ OR exp Tendons/ OR Muscles/ OR exp Muscle, Skeletal/ OR exp Muscle, Smooth/ OR exp Musculoskeletal Diseases/ OR exp Nervous System/ OR exp Nervous System Diseases/ OR exp Sense Organs/ OR exp Eye Diseases/ OR exp Otorhinolaryngologic Diseases/ OR exp Stomatognathic System/ OR exp Stomatognathic Diseases/ OR exp Urogenital System/ OR exp Male Urogenital Diseases/ OR exp Female Urogenital Diseases/ OR exp Neoplasms/ OR exp Metabolism/ OR metabolism.fx. OR exp Metabolic Diseases/ OR (organ* OR ((digestive* OR alimentar*) ADJ3 (system* OR tract* OR canal*)) OR gastrointest OR intest* OR gut OR guts OR anus OR anal OR esophag* OR oesophag* OR gastr* OR cricopharyng* OR foregut* OR rectum OR rectal OR stomach* OR colon* OR colorect* OR cecum* OR caecum* OR coecum* OR appendi* OR duoden* OR ileocec* OR ileum* OR jejunum* OR midgut* OR pancrea* OR biliar* OR bile-duct* OR gallbladder* OR adrenal OR (suprarenal* ADJ3 gland*) OR gonad* OR ovar* OR testis OR testes OR testicular* OR genital* OR gamete* OR muellerian-duct* OR hypophys* OR ((endocrine* OR neuroendocrine*) ADJ3 (system*)) OR pancrea* OR langerhans* OR paraganglion* OR parathyroid* OR thymus* OR thyroid* OR pituitar* OR adenohypophys* OR neurohypophys* OR exocrine* OR accessory-sex-gland* OR Bartholin* OR breast* OR ((bulbourethral* OR gastrointest* OR mucus* OR paraurethral* OR saliva* OR harder* OR salt* OR sebaceous* OR sweat*) ADJ3 (gland*)) OR lacrimal* OR tear OR tears OR prostat* OR ((antigen-presenting* OR endothel* OR subendothel* OR mast* OR dendritic* OR inflammator* OR polymorphonuclear* OR foam*) ADJ3 (cell*)) OR phagocyt* OR autoimmune* OR rheuma* OR integument* OR tegumkent* OR skin* OR hair* OR nail* OR ((sweat* OR apocrine* OR eccrine* OR sudorifera*) ADJ3 gland*) OR derma* OR dermis* OR epiderma* OR epidermis* OR scalp* OR wrinkle* OR cutis OR muscle* OR muscul* OR sphincter* OR tendon* OR bone* OR cartilage* OR fascia* OR joint* OR ligament* OR rib OR ribs OR skelet* OR pelvic OR pelvis OR shoulder* OR nerve* OR nervous* OR neuro* OR glia* OR motor-system* OR postganglion* OR preganglion* OR presynap* OR ganglion* OR synapse* OR spinal-cord* OR neural* OR brain* OR forebrain* OR cerebr* OR cerebell* OR meninx OR meninges OR dura OR dural OR epidural OR ((auditory OR gustatory OR vestibular OR visual OR optic*) ADJ3 system*) OR sensorium* OR neural-analy* OR sense-organ* OR neuroepithel* OR sensorimotor* OR sensory OR ear OR ears OR taste-bud OR olfactory OR eye* OR optic-pathway* OR ocul* OR opthal* OR photoreceptor* OR stomatognathic* OR mouth* OR tooth OR teeth OR dentition* OR dental* OR pharyn* OR gingiva* OR periodont* OR cheek* OR lip OR lips OR palat* OR saliva* OR tongue* OR urogenital* OR genital* OR urinar* OR cloaca* OR genitourinary* OR sex-gland* OR sex-organ* OR gamete* OR gonad* OR kidney* OR renal* OR urolog* OR cancer* OR neoplas* OR tumor* OR tumour* OR malign* OR metastas* OR carcino* OR adenocarcino* OR metabol* OR thromb* OR exp Renin/ OR exp Renin-Angiotensin System/ OR renin OR aldosterone) OR (exp Cholesterol/ OR cholesterol) OR (“sex organ*” or urogenital* or genital* or reproduct* or placent*)) NOT (exp animals/ NOT humans/) NOT (review.ti.) NOT (review OR letter* OR news OR comment* OR editorial* OR congres* OR abstract* OR book* OR chapter* OR dissertation abstract*).pt.

**Cochrane Central**

((fontan OR single NEXT ventric* OR univentric* OR ((cavopulmonar* OR cavo NEXT pulmonar*) NEAR/3 (connection* OR anastomos* OR shunt*)) OR (norwood NEAR/3 (stage NEXT 3 OR stage NEXT iii))):ab,ti,kw) AND ((organ* OR ((digestive* OR alimentar*) NEAR/3 (system* OR tract* OR canal*)) OR gastrointest OR intest* OR gut OR guts OR anus OR anal OR esophag* OR oesophag* OR gastr* OR cricopharyng* OR foregut* OR rectum OR rectal OR stomach* OR colon* OR colorect* OR cecum* OR caecum* OR coecum* OR appendi* OR duoden* OR ileocec* OR ileum* OR jejunum* OR midgut* OR pancrea* OR biliar* OR bile NEXT duct* OR gallbladder* OR adrenal OR (suprarenal* NEAR/3 gland*) OR gonad* OR ovar* OR testis OR testes OR testicular* OR genital* OR gamete* OR muellerian NEXT duct* OR hypophys* OR ((endocrine* OR neuroendocrine*) NEAR/3 (system*)) OR pancrea* OR langerhans* OR paraganglion* OR parathyroid* OR thymus* OR thyroid* OR pituitar* OR adenohypophys* OR neurohypophys* OR exocrine* OR accessory NEXT sex NEXT gland* OR Bartholin* OR breast* OR ((bulbourethral* OR gastrointest* OR mucus* OR paraurethral* OR saliva* OR harder* OR salt* OR sebaceous* OR sweat*) NEAR/3 (gland*)) OR lacrimal* OR tear OR tears OR prostat* OR ((antigen NEXT presenting* OR endothel* OR subendothel* OR mast* OR dendritic* OR inflammator* OR polymorphonuclear* OR foam*) NEAR/3 (cell*)) OR macrophage* OR leukocyte* OR phagocyt* OR autoimmune* OR rheuma* OR integument* OR tegumkent* OR skin* OR hair* OR nail* OR ((sweat* OR apocrine* OR eccrine* OR sudorifera*) NEAR/3 gland*) OR derma* OR dermis* OR epiderma* OR epidermis* OR scalp* OR wrinkle* OR cutis OR muscle* OR muscul* OR sphincter* OR tendon* OR bone* OR cartilage* OR fascia* OR joint* OR ligament* OR rib OR ribs OR skelet* OR pelvic OR pelvis OR shoulder* OR nerve* OR nervous* OR neuro* OR glia* OR motor NEXT system* OR postganglion* OR preganglion* OR presynap* OR ganglion* OR synapse* OR spinal NEXT cord* OR neural* OR brain* OR forebrain* OR cerebr* OR cerebell* OR meninx OR meninges OR dura OR dural OR epidural OR ((auditory OR gustatory OR vestibular OR visual OR optic*) NEAR/3 system*) OR sensorium* OR neural NEXT analy* OR sense NEXT organ* OR neuroepithel* OR sensorimotor* OR sensory OR ear OR ears OR taste NEXT bud OR olfactory OR eye* OR optic NEXT pathway* OR ocul* OR opthal* OR photoreceptor* OR stomatognathic* OR mouth* OR tooth OR teeth OR dentition* OR dental* OR pharyn* OR gingiva* OR periodont* OR cheek* OR lip OR lips OR palat* OR saliva* OR tongue* OR urogenital* OR genital* OR urinar* OR cloaca* OR genitourinary* OR sex NEXT gland* OR sex NEXT organ* OR gamete* OR gonad* OR kidney* OR renal* OR urolog* OR cancer* OR neoplas* OR tumor* OR tumour* OR malign* OR metastas* OR carcino* OR adenocarcino* OR metabol* OR thromb* OR renin OR aldosterone OR cholesterol OR urogenital* OR genital* OR reproduct* OR placent*):ab,ti,kw)
