# Supplementary material for: Fontan Circulation Associated Organ Abnormalities Beyond the Heart, Lungs, Liver, and Gut: A Systematic Review
Source: Front Cardiovasc Med. 2022 Mar 22;9:826096. doi: 10.3389/fcvm.2022.826096 (PMC8981209; doi:10.3389/fcvm.2022.826096)
Supplement: Supplementary file 3 [file Data_Sheet_2.docx]

**Excluded full-text articles based on consensus meeting:**

1. Abe I, Nomura M, Sakamoto R, Watanabe T, Gondo S, Ohe K, et al. Two cases of pheochromocytoma associated with single ventricle syndrome. Nippon Naika Gakkai Zasshi. 2007;96(5):994-6.

2. Ackerman LL, Kralik SF, Daniels Z, Farrell A, Schamberger MS, Mastropietro CW. Alterations in cerebral ventricle size in children with congenital heart disease. Child's Nerv Syst. 2018;34(11):2233-40.

3. Ackerman LL, Mastropietro C, Daniels Z, Kraljic S, Harris M, Farrell A, et al. Alterations in cerebral ventricle size over time in children with congenital heart disease. J Neurosurg. 2017;126(4):A1384.

4. Aguilar DC, Raff GW, Tancredi DJ, Griffin IJ. Childhood growth patterns following congenital heart disease. Cardiol Young. 2015;25(6):1044-53.

5. Aliyev B, Bayramoglu Z, Nisli K, Omeroglu RE, Dindar A. Quantification of Hepatic and Splenic Stiffness after Fontan Procedure in Children and Clinical Implications. Ultrasound Q. 2020;36(4):350-6.

6. Alstrup M, Mohanakumar S, Hjortdal V. Increased lymphatic contraction frequency in Fontan: Innate or adaptive response? A NIRF imaging study. World J Pediatr Congenit Heart Surg. 2019;10(2):NP4.

7. Avitabile C, Brodsky J, Leonard MB, Dodds K, Rush CH, Goldmuntz E, et al. Abnormalities in bone density, bone structure and muscle cross-sectional area after fontan palliation. J Am Coll Cardiol. 2013;61(10):E486.

8. Barker PC, Nowak C, King K, Mosca RS, Bove EL, Goldberg CS. Risk factors for cerebrovascular events following fontan palliation in patients with a functional single ventricle. Am J Cardiol. 2005;96(4):587-91.

9. Bédard E, Lopez S, Perron J, Houde C, Couture C, Vaillancourt R, et al. Life-threatening hemoptysis following the Fontan procedure. Can J Cardiol. 2008;24(2):145-7.

10. Bendaly EA, DiMeglio LA, Fadel WF, Hurwitz RA. Bone Density in Children with Single Ventricle Physiology. Pediatr Cardiol. 2015;36(4):779-85.

11. Binotto MA, Maeda NY, Lopes AA. Altered endothelial function following the Fontan procedure. Cardiol Young. 2008;18(1):70-4.

12. Bork KT, To BP, Leonard NJ, Douglas CM, Dinon DA, Leonard EE, et al. Prevalence of Childhood Permanent Hearing Loss after Early Complex Cardiac Surgery. J Pediatr. 2018;198:104-9.

13. Breatnach CR, Cleary A, Prendiville T, Crumlish K, Murchan H, McMahon CJ. Prevalence of Subclinical Enteric Alpha-1-Antitrypsin Loss in Children with Univentricular Circulation Following Total Cavopulmonary Connection. Pediatr Cardiol. 2018;39(1):33-7.

14. Buchhorn R, Kiessling C, Wessel A, Grunewald RW, Hufner M, Niedmann PD, et al. Neurohormonal stimulation in patients with congenital heart diseases - Influence of age, cardiac defect and hemodynamic. Herz Kreisl. 2000;32(1):22-8.

15. Buompadre MC, Arroyo HA. Accidental cerebral venous gas embolism in a young patient with congenital heart disease. J Child Neurol. 2008;23(1):121-3.

16. Burchill LJ, Redington AN, Silversides CK, Ross HJ, Jimenez-Juan L, Mital S, et al. Renin-angiotensin-aldosterone system genotype and serum BNP in a contemporary cohort of adults late after Fontan palliation. Int J Cardiol. 2015;197:209-15.

17. Burns L, Maul EC. Benign pneumatosis intestinalis in a child with cavopulmonary shunt and immune thrombocytopenic purpura. J Hosp Med. 2009;4(7):E41-E2.

18. Butera G, Bonnet D, Bonhoeffer P, Aggoun Y, Iserin L, Sidi D, et al. Alterations in the autonomic nervous system in patients with Fontan circulation: Preliminary data. G Ital Cardiol. 1999;29(3):255-60.

19. Cabrera AG, Kibler KK, Blaine Easley R, Goldsworthy M, Shekerdemian LS, Andropoulos DB, et al. Elevated arterial blood pressure after superior cavo-pulmonary anastomosis is associated with elevated pulmonary artery pressure and cerebrovascular dysautoregulation. Pediatr Res. 2018;84(3):356-61.

20. Callegari A, Christmann M, Albisetti M, Kretschmar O, Quandt D. Single Ventricle Physiology Patients and Coagulation Abnormalities: Is There a Relationship With Hemodynamic Data and Postoperative Course? A Pilot Study. Clin Appl Thromb Hemost. 2019;25.

21. Carpenter J, Keating R, Weinstein S, Vezina G, Berger J, Bell MJ. Cerebral hemorrhage and vasospasm in a child with congenital heart disease. Neurocrit Care. 2008;8(2):276-9.

22. Chaloupecký V, Svobodová I, Hadaćova I, Tomek V, Hučín B, Tláskal T, et al. Coagulation profile and liver function in 102 patients after total cavopulmonary connection at mid term follow up. Heart. 2005;91(1):73-9.

23. Cheng HH, Carmona F, McDavitt E, Wigmore D, Perez-Rossello JM, Gordon CM, et al. Fractures Related to Metabolic Bone Disease in Children with Congenital Heart Disease. Congenit Heart Dis. 2016;11(1):80-6.

24. Cheng HH, Rajagopal S, McDavitt E, Wigmore D, Williams K, Thiagarajan R, et al. Stroke in Acquired and Congenital Heart Disease Patients and Its Relationship to Hospital Mortality and Lasting Neurologic Deficits. Pediatr Crit Care Med. 2016;17(10):976-83.

25. Cheung EWY, Chay GW, Ma ESK, Cheung YF. Systemic oxygen saturation and coagulation factor abnormalities before and after the fontan procedure. Am J Cardiol. 2005;96(11):1571-5.

26. Chun DS, Schamberger MS, Flaspohler T, Turrentine MW, Brown JW, Farrell AG, et al. Incidence, outcome, and risk factors for stroke after the Fontan procedure. Am J Cardiol. 2004;93(1):117-9.

27. Cohen S, Gurvitz MZ, Beauséjour-Ladouceur V, Lawler PR, Therrien J, Marelli AJ. Cancer Risk in Congenital Heart Disease-What Is the Evidence? Can J Cardiol. 2019;35(12):1750-61.

28. Collins N, Piran S, Harrison J, Azevedo E, Oechslin E, Silversides CK. Prevalence and Determinants of Anemia in Adults With Complex Congenital Heart Disease and Ventricular Dysfunction (Subaortic Right Ventricle and Single Ventricle Physiology). Am J Cardiol. 2008;102(5):625-8.

29. Cordina R, Grieve S, Barnett M, Lagopoulos J, Malitz N, Celermajer DS. Brain Volumetrics, Regional Cortical Thickness and Radiographic Findings in Adults with Cyanotic Congenital Heart Disease. NeuroImage Clin. 2014;4:319-25.

30. Cromme-Dijkhuis AH, Henkens CMA, Bijleveld CMA, Hillege HL, Bom VJJ, Van der Meer J. Coagulation factor abnormalities as possible thrombotic risk factors after Fontan operations. LANCET. 1990;336(8723):1087-90.

31. Cromme-Dijkhuis AH, Hess J, Hahlen K, Henkens CMA, Bink-Boelkens MTE, Eygelaar AA, et al. Specific sequelae after Fontan operation at mid- and long-term follow-up: Arrhythmia, liver dysfunction, and coagulation disorders. J THORAC CARDIOVASC SURG. 1993;106(6):1126-32.

32. Day RW, Boyer RS, Tait VF, Ruttenberg HD. Factors associated with stroke following the Fontan procedure. PEDIATR CARDIOL. 1995;16(6):270-5.

33. Drenthen W, Hoendermis ES, Moons P, Heida KY, Roos-Hesselink JW, Mulder BJM, et al. Menstrual cycle and its disorders in women with congenital heart disease. Congenit Heart Dis. 2008;3(4):277-83.

34. Du Plessis AJ, Chang AC, Wessel DL, Lock JE, Wernovsky G, Newburger JW, et al. Cerebrovascular accidents following the Fontan operation. PEDIATR NEUROL. 1995;12(3):230-6.

35. Egbe AC, Connolly HM, Niaz T, Yogeswaran V, Taggart NW, Qureshi MY, et al. Prevalence and outcome of thrombotic and embolic complications in adults after Fontan operation. Am Heart J. 2017;183:10-7.

36. Fontes K, Rohlicek CV, Saint-Martin C, Gilbert G, Easson K, Majnemer A, et al. Hippocampal alterations and functional correlates in adolescents and young adults with congenital heart disease. Hum Brain Mapp. 2019;40(12):3548-60.

37. François K, Bové T, De Groote K, Panzer J, Vandekerckhove K, Suys B, et al. Pleural effusions, water balance mediators and the influence of lisinopril after completion Fontan procedures. Eur J Cardio-thorac Surg. 2009;36(1):57-62.

38. Fujii T, Shimizu T, Takahashi K, Kishiro M, Ohkubo M, Akimoto K, et al. Fecal alpha1-antitrypsin concentrations as a measure of enteric protein loss after modified fontan operations. J Pediatr Gastroenterol Nutr. 2003;37(5):577-80.

39. Furuse A, Koseni K, Takeshita M, Kotsuka Y, Yagyu K, Kawauchi M. Retrograde gastric varices in a patient with total cavopulmonary shunt. ANN THORAC SURG. 1993;55(6):1574-5.

40. Garty BZ. Deficiency of CD4+ lymphocytes due to intestinal loss after Fontan procedure [1]. Eur J Pediatr. 2001;160(1):58-9.

41. Gissel M, Tomkiewicz-Pajak L, Podolec P, Hoffman P, Trojnarska O, Lipczyńska M, et al. In silico thrombin and factor Xa generation profiles in adult patients after Fontan operation. Blood Coagul Fibrinolysis. 2018;29(2):236-40.

42. Glöckler M, Severin T, Arnold R, Greiner P, Schwab KO, Uhl M, et al. First description of three patients with multifocal lymphangiomatosis and protein-losing enteropathy following palliation of complex congenital heart disease with total cavo-pulmonary connection. Pediatr Cardiol. 2008;29(4):771-4.

43. Goldberg DJ, Dodds K, Avitabile CM, Glatz AC, Brodsky JL, Semeao EJ, et al. Children with protein-losing enteropathy after the Fontan operation are at risk for abnormal bone mineral density. Pediatr Cardiol. 2012;33(8):1264-8.

44. Güçer Ş, Ince T, Kale G, Akçdsoren Z, Özkutlu S, Talim B, et al. Noncardiac malformations in congenital heart disease: A retrospective analysis of 305 pediatric autopsies. Turk J Pediatr. 2005;47(2):159-66.

45. Guimarães VA, Castelli JB, Atik E. Rare complications after Fontan operation. Cardiol Young. 2010;20(4):442.

46. Haarman MG, Vos JDL, Berger RMF, Willems TP, Jeneson JAL. Failing Homeostasis of Quadriceps Muscle Energy- and pH Balance During Bicycling in a Young Patient With a Fontan Circulation. Front Cardiovasc Med. 2019;6.

47. Halaweish I, Ralls M, Siddiqui S, Dickinson C, Jarboe MD. Obstructive jaundice secondary to Morgagni hernia in an infant with Fontan circulation. Pediatr Surg Int. 2015;31(12):1199-202.

48. Hayashi T, Inuzuka R, Shindo T, Hirata Y, Shimizu N, Oka A. Serum hyaluronic acid concentration in Fontan circulation: Correlation with hepatic function and portal vein hemodynamics. Pediatr Cardiol. 2014;35(4):608-15.

49. Hayashida K, Hirose Y, Kume N, Nishioeda Y, Ihn Ho C, Shimotsu Y, et al. Postural cerebral hypoperfusion related to brain atrophy after a modified fontan operation. Clin Nucl Med. 1998;23(8):539-40.

50. Hlavackova E, Liska M, Jicinska H, Navratil J, Litzman J. Secondary combined immunodeficiency in pediatric patients after the Fontan operation: Three case reports. Int Arch Allergy Immunol. 2016;170(4):251-6.

51. Horigome H, Iwasaki N, Anno I, Kurachi S, Kurachi K. Magnetic resonance imaging of the brain and haematological profile in adult cyanotic congenital heart disease without stroke. Heart. 2006;92(2):263-5.

52. Ikpot IZ, Reeves K, Awad H. Is Electroconvulsive Therapy Safe after a Fontan Repair? J ECT. 2021;37(2):e21-e2.

53. Jahangiri M, Shore D, Kakkar V, Lincoln C, Shinebourne E, Bailey LL, et al. Coagulation factor abnormalities after the fontan procedure and its modifications. J THORAC CARDIOVASC SURG. 1997;113(6):989-93.

54. Kajimoto H, Nakazawa M, Murasaki K, Hagiwara N, Nakanishi T. Increased P-selectin expression on platelets and decreased plasma thrombomodulin in Fontan patients. Circ J. 2009;73(9):1705-10.

55. Kaulitz R, Haber P, Sturm E, Schäfer J, Hofbeck M. Serial evaluation of hepatic function profile after Fontan operation. Herz. 2014;39(1):98-104.

56. Kaulitz R, Luhmer I, Bergmann F, Rodeck B, Hausdorf G. Sequelae after modified Fontan operation: Postoperative haemodynamic data and organ function. HEART. 1997;78(2):154-9.

57. Khalil A, Trehan R, Tiwari A, Malik R, Arora R. Immunological profile in congenital heart disease. Indian Pediatr. 1994;31(3):295-300.

58. Knirsch W, Häusermann E, Fasnacht M, Hersberger M, Gessler P, Bauersfeld U. Plasma B-type natriuretic peptide levels in children with heart disease. Acta Paediatr Int J Paediatr. 2011;100(9):1213-6.

59. Koch A, Zink S, Singer H. B-type natriuretic peptide in paediatric patients with congenital heart disease. Eur Heart J. 2006;27(7):861-6.

60. Koch AME, Zink S, Singer H, Dittrich S. B-type natriuretic peptide levels in patients with functionally univentricular hearts after total cavopulmonary connection. Eur J Heart Fail. 2008;10(1):60-2.

61. Lambrecht L, Cools B, Witters P. A rare cause of persisting anaemia in a patient with a failing Fontan circulation. Cardiol Young. 2017;27(1):176-7.

62. Malloy L, Jensen M, Bishop W, Divekar A. "Downhill" esophageal varices in congenital heart disease. J Pediatr Gastroenterol Nutr. 2013;56(2):e9-e11.

63. Mehrotra A, Khanna P, Kumar S, Abraham G. Renal abscess after the Fontan procedure: A case report. J Med Case Rep. 2011;5.

64. Morchi GS, Wolfe RR, Kay JD. Antiphospholipid syndrome leading to myocardial infarction in a fontan patient 17 years after norwood palliation. Congenit Heart Dis. 2009;4(1):54-8.

65. Poh C, Hornung T, Celermajer DS, Radford DJ, Justo RN, Andrews D, et al. Modes of late mortality in patients with a Fontan circulation. Heart. 2020;106(18):1427-31.

66. Puri K, Price JF, Spinner JA, Powers JM, Denfield SW, Cabrera AG, et al. Iron Deficiency Is Associated with Adverse Outcomes in Pediatric Heart Failure. J Pediatr. 2020;216:58-66.e51.

67. Santoro G, Carrozza M, Bigazzi MC, Morelli C, Sessa F, Calabrò R. Life-threatening hemoptysis after the Fontan procedure. Ital Heart J. 2003;4(2):139-41.

68. Sosa Lozano LA, Shahir K, Akbar M, Goodman LR. A case of tracheal varices: An unusual but important cause of mural nodules in the trachea. Br J Radiol. 2011;84(999):e62-e4.

69. Thorne SA, Hooper J, Kemp M, Somerville J. Gastro-intestinal protein loss in late survivors of Fontan surgery and other congenital heart disease. Eur Heart J. 1998;19(3):514-20.

70. Tomkiewicz-Pajak L, Plazak W, Kolcz J, Pajak J, Kopec G, Dluzniewska N, et al. Iron deficiency and hematological changes in adult patients after Fontan operation. J Cardiol. 2014;64(5):384-9.

71. Udink Ten Cate FEA, Hannes T, Broekaert I, Hünseler C, Brockmeier K, Sreeram N. Small intestinal bacterial overgrowth in fontan patients with protein-losing enteropathy: Preliminary results. Thorac Cardiovasc Surg. 2016;64.

72. Udink Ten Cate FEA, Nazzal R, Hannes T, Broekaert I, Hünseler C, Brockmeier K, et al. Small intestinal bacterial overgrowth in Fontan patients with protein-losing enteropathy: Preliminary results. Cardiol Young. 2016;26:S170-S1.

73. Wolff D, Ebels T, Van Melle JP. N-terminal pro brain natriuretic hormone in Fontan patients: Heart failure or circulatory failure? Eur J Heart Fail. 2013;15(6):602-3.

74. Yamagata Y, Kawano H, Shibata H, Maemura K. Esophageal varix in an adult fontan survivor. Intern Med. 2017;56(23):3261-2.

75. Yoshikawa Y, Uemura H, Yagihara T, Kawahira Y, Ohuchi H, Kitamura S. Functional status in adolescents and adults with Fontan circulation. Jpn J Thorac Cardiovasc Surg. 2002;50(4):141-5.

76. Zentner D, Kotevski A, King I, Grigg L, D'Udekem Y. Fertility and pregnancy in the Fontan population. International Journal of Cardiology. 2016;208((Zentner D., dominica.zentner@mh.org.au; Grigg L.) Department of Cardiology, Royal Melbourne Hospital, VIC, Australia):97-101.

77. Guntheroth WG. The fontan procedure and pregnancy. ACC Current Journal Review. 1999;8(1):98.
